# Untangling the origin and function of granulovacuolar degeneration bodies in neurodegenerative proteinopathies

**DOI:** 10.1186/s40478-020-00996-5

**Published:** 2020-09-03

**Authors:** Vera I. Wiersma, Jeroen J. M. Hoozemans, Wiep Scheper

**Affiliations:** 1 Department of Clinical Genetics, Amsterdam University Medical Centers location VUmc, Amsterdam, The Netherlands; 2 Department of Functional Genomics, Center for Neurogenomics and Cognitive Research, Vrije Universiteit (VU), De Boelelaan 1085, 1081 HV Amsterdam, The Netherlands; 3 Department of Pathology, Amsterdam University Medical Centers location VUmc, Amsterdam, The Netherlands

**Keywords:** Granulovacuolar degeneration bodies, Tau pathology, Neurodegenerative proteinopathies, Lysosomes

## Abstract

In the brains of tauopathy patients, tau pathology coincides with the presence of granulovacuolar degeneration bodies (GVBs) both at the regional and cellular level. Recently, it was shown that intracellular tau pathology causes GVB formation in experimental models thus explaining the strong correlation between these neuropathological hallmarks in the human brain. These novel models of GVB formation provide opportunities for future research into GVB biology, but also urge reevaluation of previous post-mortem observations. Here, we review neuropathological data on GVBs in tauopathies and other neurodegenerative proteinopathies. We discuss the possibility that intracellular aggregates composed of proteins other than tau are also able to induce GVB formation. Furthermore, the potential mechanisms of GVB formation and the downstream functional implications hereof are outlined in view of the current available data. In addition, we provide guidelines for the identification of GVBs in tissue and cell models that will help to facilitate and streamline research towards the elucidation of the role of these enigmatic and understudied structures in neurodegeneration.

Neurodegenerative tauopathies, including Alzheimer’s disease (AD) and frontotemporal lobar degeneration (FTLD) share a common neuropathological feature: the deposition of misfolded and hyperphosphorylated tau protein in multimeric, highly-ordered, filamentous aggregates in the central nervous system. The existence of familial forms of FTLD that are caused by mutations in the tau-encoding *MAPT* gene underscores the key role of tau in disease pathogenesis [49, 109, 121]. Indeed, also in sporadic tauopathy patients the accumulation of aggregated tau strongly correlates with neuronal loss and clinical symptoms, as demonstrated by neuropathological studies [4, 34, 51, 98] and more recently in living patients by tau positron emission tomography [77]. Despite this strong association between tau aggregation and neurodegeneration, the mechanisms that connect the two are largely unknown. Here, we focus on an intracellular alteration that coincides with the formation of tau aggregates: the emergence of granulovacuolar degeneration bodies (GVBs).

GVBs were first observed in 1911 by Simchowicz, in pyramidal neurons in the hippocampus of AD patients [120]. GVBs are membrane-delineated clear vacuoles that harbor a dense core or “granule”. The GVB vacuole and core measure between ~ 3 to ~ 5 μm and ~ 0.5 and ~ 1.5 μm in diameter, respectively. The number of GVBs per cell is highly variable, ranging from a single one to dozens. They are predominantly found in the neuronal cell body but can also be localized in the (proximal) neurite. Based on the basophilic and argentophilic properties of the core, GVBs have traditionally been visualized using the routine histological hematoxylin and eosin (H&E) staining and by silver impregnation techniques [133]. Currently, GVBs are most commonly detected by immunolabeling of the core using specific markers (Table 1; Fig. 1). The presence of GVBs within cells constitutes the neuropathological hallmark known as granulovacuolar degeneration (GVD).

**Table 1:** *Box:* Validation of GVB identity

Both the core and membrane of GVBs carry epitopes that can be used to confirm a GVB identity. Commonly used GVB core markers that consistently detect GVBs in human brain and experimental models are CK1δ (Fig. 1a-d) and CK1ε [33, 67, 143], CHMP2B [90, 143, 147], pPERK (Fig. 1c-f), peIF2α and pIRE1α [43, 67, 143] and pTDP-43 [47, 59, 79, 148]. In Supplementary Table 1, an overview of primary antibodies used to detect these common GVB markers in tissue and culture is provided. Of these common GVB markers, the evidence for the localization of CK1 isoforms to human and experimental GVBs meets high standards of rigor. The presence of CK1δ and CK1ε in human and experimental GVBs has been shown using antibodies that stain GVBs in a manner independent of phosphorylation – as phosphatase pre-treatment of human brain tissue did not affect GVB immunolabeling [33]. When comparing CK1δ to CK1ε staining in aged tau Tg mice, a high degree of overlap was found: in ~ 40% of GVB-bearing neurons, all GVBs were double immunopositive, with the great majority of remaining neurons showing between 50 and 99% of double-labeled GVBs [67]. In addition, CK1δ and CK1ε staining overlap with GVBs detected by H&E staining in serial brain sections [33]. Furthermore, using STED super-resolution microscopy and/or immuno-EM the subcellular localization of CK1δ [33, 143] and CK1ε [74] in the GVB core has been confirmed at high resolution. Moreover, fluorescently-tagged CK1δ localizes to GVBs in vitro [143]. Therefore, CK1δ is currently the only constituent of the GVB core of which the presence has been confirmed directly, without the use of antibodies. In 100% [90]/98% [30] of CK1δ-positive neurons in the human brain, GVBs were also labeled by CHMP2B. On the subcellular level, CK1δ and CHMP2B co-localize in the core of 82% of GVBs in the human brain [30]. The overlap between the two GVB markers was somewhat lower in aged tau Tg mice, with CHMP2B immunolabeling being detected in 57% of neurons with CK1δ-positive GVBs and within those neurons 63% of GVBs being double positive for both CHMP2B and CK1δ [90]. In the human brain, CHMP2B staining overlaps with H&E staining after ethanol-mediated de-staining [146, 147] and mirrors H&E staining of GVBs in adjacent sections [147]. Furthermore, GVB counts based on CHMP2B and H&E staining strongly correlate [147]. Importantly, in cultured neurons CHMP2B does not only stain GVBs, but also yields a punctate staining pattern in control cells [143]. Therefore, in vitro GVB detection using CHMP2B requires co-staining with an additional GVB marker. Also the UPR activation markers pPERK, peIF2α and pIRE1α localize to structures that are morphologically clearly recognizable as GVBs in the human and tau Tg mouse brain [43, 67, 82, 99] (Fig. 1). The localization of pPERK to human GVBs was also shown by immuno-EM [82]. pPERK immunolabeling highly co-localizes with CK1δ-positive GVBs in aged tau Tg mice: in ~ 55% of neurons, all GVBs were double-positive for both markers – which is a higher percentage than found for the co-localization between CK1δ and CK1ε (see above) in single GVBs – and in the other ~ 45% of neurons between 50 and 99% of GVBs were positive for both CK1δ and pPERK. Also in the human brain and cultured neurons, stainings for the UPR activation markers overlap with CK1δ in GVBs [143] (Fig. 1), although the exact percentages of co-localization remain to be quantified. In conclusion, pPERK, peIF2α and pIRE1α are adequate markers of human and mouse GVBs. The Thal GVD neuropathological staging system [132] is based on immunopositivity for CK1δ and CK1ε and in addition pTDP-43 [59]. pTDP-43 localizes to structures that morphologically resemble GVBs in the human [59, 79, 132] and mouse brain [148], but has so far not been tested in the in vitro GVB model. pTDP-43 staining in tissue overlaps with CK1δ- and CK1ε-immunolabeled GVBs [132, 148], although the co-localization was not quantified. However, in the human brain the distribution pattern of GVBs is similar when determining the Thal GVD stage using CK1δ, CK1ε or pTDP-43 [132]. Furthermore, no difference was found when quantifying the percentage of GVB-positive neurons in the human brain using antibodies against CK1δ or the newly discovered GVB-localizing protein pMLKL of which the staining pattern overlaps with CK1δ- and pTDP-43-positive GVBs [68]. This indicates that also pMLKL is a suitable GVB marker in the human brain, whereas its use in experimental models remains to be validated. Although pPERK, peIF2α, pIRE1α and pTDP-43 (and pMLKL) are detected in GVBs using phospho-specific rather than generic antibodies which could interfere with their functional interpretation (Table 3), they are reliable and consistent GVB markers across tissue from different species and in experimental models. The GVB membrane that surrounds the core and vacuole is positive for LAMP1 in tissue and cells [30, 143] and can alternatively be detected using LIMP2 [143]. Although the global marker profile applies to most GVBs, future studies should characterize in more detail whether differential marker positivity of individual cores identifies different GVB structures and functions. Based on the existing literature on GVBs in post-mortem tissue and experimental models, we propose refinement of the criteria for validation of GVB identity in research settings, in order to systematize future post-mortem and experimental studies.	
In the context of diagnostics, GVBs are typically detected by H&E staining. For research purposes, we here propose refinement of the criteria to identify GVBs to aid future studies (Fig. 1). These guidelines are of use for the validation of GVB identity in tissue and cell models. We strongly recommend that the presence of a CK1δ-positive core is a prerequisite to classify an organelle as GVB (Fig. 1a, b) as CK1δ is one of the most consistent and rigorously validated GVB core markers in tissue and cells and its GVB localization is confirmed in an antibody-independent manner. In addition, immunopositivity of the CK1δ-labeled GVB core for at least one of the other common GVB core markers (pPERK, peIF2α, pIRE1α, CK1ε, pTDP-43 and CHMP2B) is recommended, demonstrated preferably by double immunolabeling (Fig. 1c, d) or by staining of adjacent tissue sections. In addition, confirmation of the distinctive GVB morphology, including the presence of the GVB membrane that surrounds core and vacuole, is recommended. In cells as well as tissue, this can be done by visualization of the GVB membrane by immunodetection of the lysosomal membrane protein LAMP1 (Fig. 1f) or LIMP2. Alternatively, this staining could be replaced by morphological confirmation of membrane, core and vacuole on H&E staining. In addition, the GVB membrane often becomes visible upon the chromogenic peroxidase-catalyzed detection of immunolabeling with a GVB core marker – e.g. by means of the widely used chromogen 3,3′-diaminobenzidine (DAB) –, whereas upon fluorescent detection only the GVB core is shown (compare pPERK immunoreactivity in Fig. 1e with Fig. 1c, d, f). This may be due to deposition of the diffusible chromogenic product at membranes during the enzymatic signal amplification step. Therefore, as DAB signal may incorrectly suggest localization of a protein to both the GVB core and membrane, fluorescent double labeling is advised to determine the subcellular localization of GVB proteins. Lastly, super-resolution microscopy and EM can be employed to study the morphology of immunolabeled GVBs at high resolution. In conclusion, we recommend three criteria for the assessment of GVB identity that includes immunoreactivity for CK1δ (criterion 1) and an additional common GVB marker (criterion 2) in the GVB core and visualization of GVB membrane and/or vacuole (criterion 3) (Fig. 1).

**Fig. 1:**
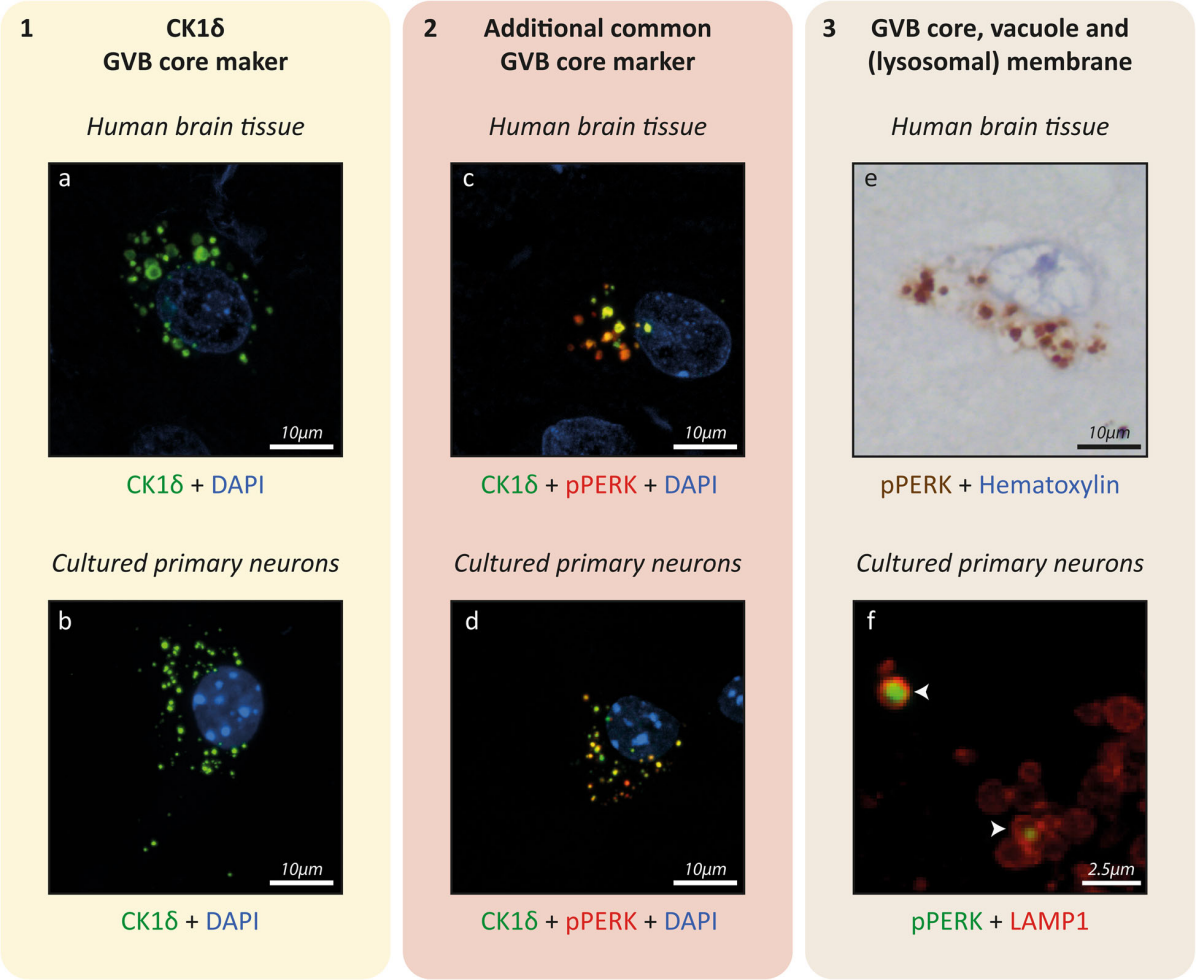
Recommendations for the identification of GVBs in a research setting: three criteria for the validation of GVB identity in tissue and cell models. Criterion 1: Immunoreactivity for the common GVB marker CK1δ in the core (tissue **a**, cells **b**). Criterion 2: Immunoreactivity of the CK1δ-positive core for another common GVB core marker determined by double immunolabeling (tissue **c**, cells **d**). Alternatively, staining of adjacent sections may be performed in experiments on tissue. Additional common GVB core markers are pPERK, peIF2α, pIRE1α, CK1ε, CHMP2B and pTDP-43. Note that CHMP2B does not exclusively stain GVBs in cultured cells and that pTDP-43 has not been tested in the in vitro model. Criterion 3: Visualization of characteristic GVB morphology, including the presence of the GVB membrane and/or vacuole (tissue **e**, cells **f**). The GVB membrane is preferably visualized by immunodetection of the lysosomal membrane marker LAMP1 (**f**) or LIMP2. In tissue, GVB morphology may also be visualized employing the routine H&E staining that is used for diagnostics. Alternatively, chromogenic peroxidase-catalyzed immunodetection of a GVB core marker often reveals GVB core, vacuole and membrane (**e**). These criteria are also of use for the validation of novel GVB markers that can be investigated by examining their co-localization with CK1δ (criterion 1) and an additional common GVB marker (criterion 2) as well as their subcellular localization to GVB core/membrane (criterion 3). **a**, **c** and **e** show immunolabeling of human AD hippocampus. **b**, **d** and **f** show immunolabeling of cultured primary mouse neurons with seeded tau pathology [143]. Cell nuclei are stained with 4′,6-diamidino-2-phenylindole (DAPI) in **a**-**d** and with hematoxylin in **e.** In **a**-**d** and **f** immunofluorescence and in **e** immunohistochemistry using the chromogen 3,3′-diaminobenzidine (DAB) was performed. Arrowheads in **f** point to GVBs. See text for details

Since their discovery over a century ago, GVBs have been enigmatic structures of unknown origin and function. In 1980, a review on senile dementia stated: “In the absence of an experimental model for the induction of granulovacuolar change, this characteristic cytological change has been largely ignored, and at the present time no hypothesis for induction is available” [11]. It took 4 more decades to the publication of the first experimental model for GVB formation in vitro, in which GVBs are induced by the seeding of intracellular tau pathology [143]. This opens new avenues for future research into the mechanism and function of GVB formation. However, the causal relation between tau pathology and GVBs also urges reevaluation of previous neuropathological observations. Here, we outline the neuropathological and experimental data connecting tau pathology and GVBs and discuss the possibility that other protein aggregates can similarly elicit GVB formation. In addition, the potential mechanisms and functional consequences of GVB formation are discussed. Furthermore, we have formulated recommendations for the validation of GVB identity in tissue and cell models to facilitate future research.

## GVBs as pathological companion of tau pathology

GVBs have been most extensively studied in the brains of AD patients. The amount of neurons with GVBs is higher in the hippocampus of AD patients compared to age-matched controls [8, 9, 43, 68, 125, 144, 145]. Although GVBs are most often detected in hippocampal pyramidal neurons, GVBs are also found beyond the hippocampal formation. The distribution of GVBs follows a stereotypical pattern through the brain, described by a score of 1–5 according to the Thal GVD neuropathological staging system [132]. In Thal GVD stages 2–5, GVBs are also detected outside of the hippocampus: first in the entorhinal cortex, then in the temporal neocortex, hypothalamus and amygdala and eventually also in frontal and parietal cortical areas. The Thal GVD stage of AD cases is significantly higher than that of age-matched controls [68, 131, 132], indicating that in AD patients more brain regions are affected by GVBs. Therefore, the GVB load in AD patients is increased both within and outside of the hippocampus.

A striking correlation between GVBs and the presence of AD-related tau pathology is observed in the human brain. In AD patients, the hippocampal formation is the hotspot for tau pathology as well as for GVBs. Within the hippocampus, the number of neurons with GVBs significantly correlates with the number of neurons with tau pathology [43]. In accordance, the hippocampal GVB load in AD patients increases with the Braak stage for neurofibrillary tangle (NFT) pathology (NFT Braak stage [12, 13]) in which the severity of tau pathology in the hippocampus increments per stage [32, 43, 69, 81, 145]. Therefore, a strong positive correlation exists between the local tau pathology and GVB load in the AD hippocampus. Furthermore, the distribution pattern of GVBs in the human brain [132] roughly follows that of AD-type tau pathology [12, 13]: both lesions first occur in and around the hippocampal formation before spreading to other limbic and neocortical areas. Hence, GVBs and AD-related tau pathology follow a similar spatiotemporal distribution pattern in the human brain. In line with this, the Thal GVD and NFT Braak stage significantly correlate [68, 132]. Moreover, GVBs and tau pathology co-localize at the single cell level in the AD brain: GVBs are typically detected in neurons that also exhibit tau pathology [5, 43, 72, 81, 90, 111, 122, 135, 146]. Therefore, GVBs and tau pathology co-occur in the same brain regions and in the same cells in AD.

The strong association between GVBs and tau pathology extends beyond AD. Also in patients with the primary tauopathy progressive supranuclear palsy (PSP), a significant increase in the number of neurons with GVBs is found in different brain areas affected by tau pathology, including the brainstem and midbrain [125]. This is in line with various descriptive studies reporting the presence of GVBs in these disease-specific brain areas in PSP patients [16, 99, 118, 123]. Similar to what has been described for AD, tau pathology is also detected in the majority of GVB-bearing cells in the pons of PSP patients [125]. Furthermore, the amount of neurons with GVBs was found to be significantly higher in patients with the primary tauopathy Pick’s disease compared to controls in the hippocampal area, one of the brain regions affected in Pick’s disease [144]. Indeed, several studies have shown that GVBs and Pick bodies co-occur in the hippocampus of Pick’s disease cases, sometimes in the same neurons [74, 94, 99, 111, 118, 134], in the absence [111, 118, 134] or only low abundant presence [74] of AD-related NFTs, indicating a specific association of Pick’s type tau pathology with GVBs. Hence, in addition to AD, also in primary tauopathy patients, the GVB frequency is increased and correlates with the presence of tau pathology.

Descriptive studies have additionally reported the presence of GVBs in the brains of patients with many other disorders that present with tau pathology. This includes cases with FTLD caused by the *MAPT* mutations G272V, P301L, L315R and P364S [97, 99, 124], pallido-ponto-nigral degeneration (PPND, caused by the N279K *MAPT* mutation) [118], corticobasal degeneration (CBD) [24, 105], argyrophilic grain disease [132], parkinsonism dementia complex of Guam [39, 40, 83, 118, 136], Down syndrome [18, 55, 118, 126], pantothenate kinase-associated neurodegeneration (previously known as Hallervorden-Spatz syndrome) [26], diffuse neurofibrillary tangles with calcification [151], myotonic dystrophy subtypes [96, 115, 116], the lysosomal storage disorders Niemann-Pick disease type C [127] and Salla disease [6], normal pressure hydrocephalus [10], tuberous sclerosis [41], subacute sclerosing panencephalitis [86], meningioangiomatosis [106] and various type of brain tumors [15, 56, 71, 106, 110]. Therefore, independent of disease etiology, tau pathology and GVBs coincide in the diseased human brain.

Interestingly, although the hippocampal GVB burden increases with age in subjects without clinical symptoms [9, 31, 125, 144], GVBs are also observed in the brains of young patients with tau pathology. For example, GVBs were found to accompany tau pathology/argentophilic NFTs in a 13-year-old patient with tuberous sclerosis [41], a 15-year-old patient with subacute sclerosing panencephalitis [86], a 15-year-old patient with Down syndrome [55], 34- and 41-year-old patients with different lysosomal storage disorders [6, 127] and young patients with Fukuyama-type congenital muscular dystrophy (age range: 14–34 years) [115]. This is further corroborated by the finding of GVBs in neoplastic neurons with tau pathology in patients with ganglion cell tumors (mean age: 44 years), whereas neither GVBs nor tau pathology were detected in adjacent normal brain tissue in the same patients [15]. Also in cortical neurons entrapped by meningioangiomatosis – a benign tumor-like lesion/mass – GVBs and argentophilic NFTs co-occur in a subset of cases, including a 17-year-old patient [106]. Therefore, the concurrence of tau pathology and GVBs in the human brain is independent of age.

A few studies have reported GVBs in tau transgenic (Tg) mouse models. GVBs are found in the brains of aged tau Tg mice expressing human mutant tau – more specifically, in mice from the strains JNPL3 [54, 78] and pR5 [64, 66, 67, 90, 148] expressing human tau P301L (Table 2). In contrast, GVBs are not or only rarely found in age-matched non-Tg littermates [54, 64, 66, 67, 90]. Similar to what has been documented for the human brain, the GVB load in brains of tau Tg mice increases with age in parallel with the extent of tau pathology [66, 67, 90] and follows a similar regional distribution pattern as the tau pathology [67, 90]. Furthermore, at the cellular level tau pathology co-exists in the majority of neurons with GVBs [67, 90]. Therefore, studies on tau Tg mice show that GVBs arise in parallel with tau pathology as a result of mutant tau overexpression. However, in these mouse models, GVBs are low abundant and require at least 8.5 months of transgene overexpression [54, 66, 67, 78, 90] (Table 2). This scarcity of GVB formation in tau Tg models limits their use for research into the etiology of GVBs and their role in pathology. Putative GVBs were also observed in different experimental rat models [1, 46, 138], an aged wolverine [114], 5 aged cynomolgus monkeys [22], 2 aged rat [52] and in a few cellular models [63, 88, 91]. However, the structures observed in these studied await confirmation of GVB identity using specific GVB markers (Table 1; Fig. 1; Table 2). Nonetheless, it is interesting to note that in most of these studies pathological changes in tau [22, 88, 91, 114, 138] or senile changes resembling NFTs [1, 46] are also described.

**Table 2:**
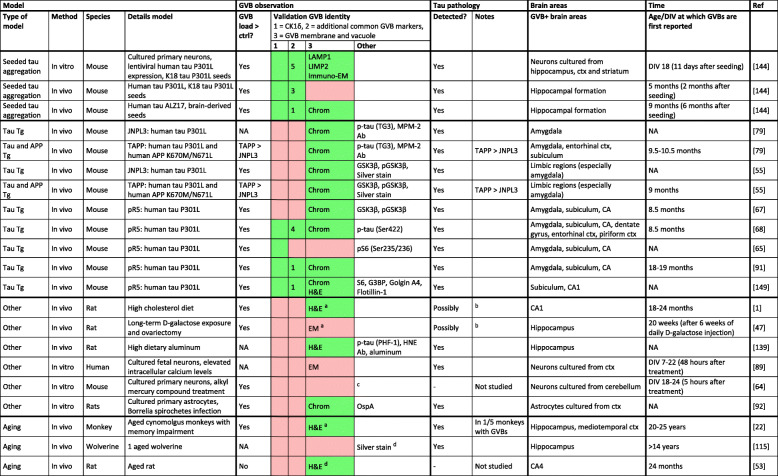
Putative GVB observations in experimental models and aged animals

Model: type of model, method, studied species and details of the model are listed. GVB observation: for experiments in which a control group was included, it is indicated whether the GVB load was higher in the experimental/symptomatic/aged group than in the control/asymptomatic/younger group. Color-coding is used to denote if studies meet the criteria for validation of GVB identity (Table 1; Fig. 1). *Green*: criterion met; *red*: criterion not met. For criterion 2, the number of additional common GVB markers used in the study is noted. For criterion 3, the method used to meet this criterion is indicated. Note that the detection of putative GVBs by EM without immunolabeling is not sufficient to meet criterion 3. Other antibodies and methods used to probe GVBs are listed, with antibody names or phospho-epitopes shown in brackets. Tau pathology: the detection of tau pathology in the animals/cultures and additional notes hereon are listed. Brain areas with GVB detection (Brain areas) and the earliest age or DIV at which GVBs were reported (Time) are shown. Reference to publications is included (Ref). ^*a*^ Putative GVBs (possibly) detected in nucleus rather than cytoplasm. ^*b*^ NFT-like structures were seen by H&E staining or on EM, but no anti-tau antibodies or silver staining were used. ^*c*^ Putative GVBs were observed by light microscopy without labeling. ^*d*^ No typical example of putative GVBs shown. Note that for clarity models of Aβ amyloidosis and aged non-Tg littermates of tau Tg mice are not included in this table

Abbreviations: *Ab* antibody, *CA* cornu ammonis, *Chrom* GVB morphology visualized by chromogenic peroxidase-catalyzed immunodetection of GVB core marker, *ctrl* control(s), *ctx* cortex, *DIV* days in vitro, *GVB* granulovacuolar degeneration body, *GVB+* GVB-containing, *EM* electron microscopy without immunodetection of GVB marker, *H&E* hematoxylin and eosin, *Immuno-EM* electron microscopy with immunodetection of GVB marker, *NA* not available, *p* phosphorylated, *Ref* reference, *Tg* transgenic, *>* higher than, *-* not applicable. For abbreviations of protein names and antibodies see “List of abbreviations”

**Fig. 2:**
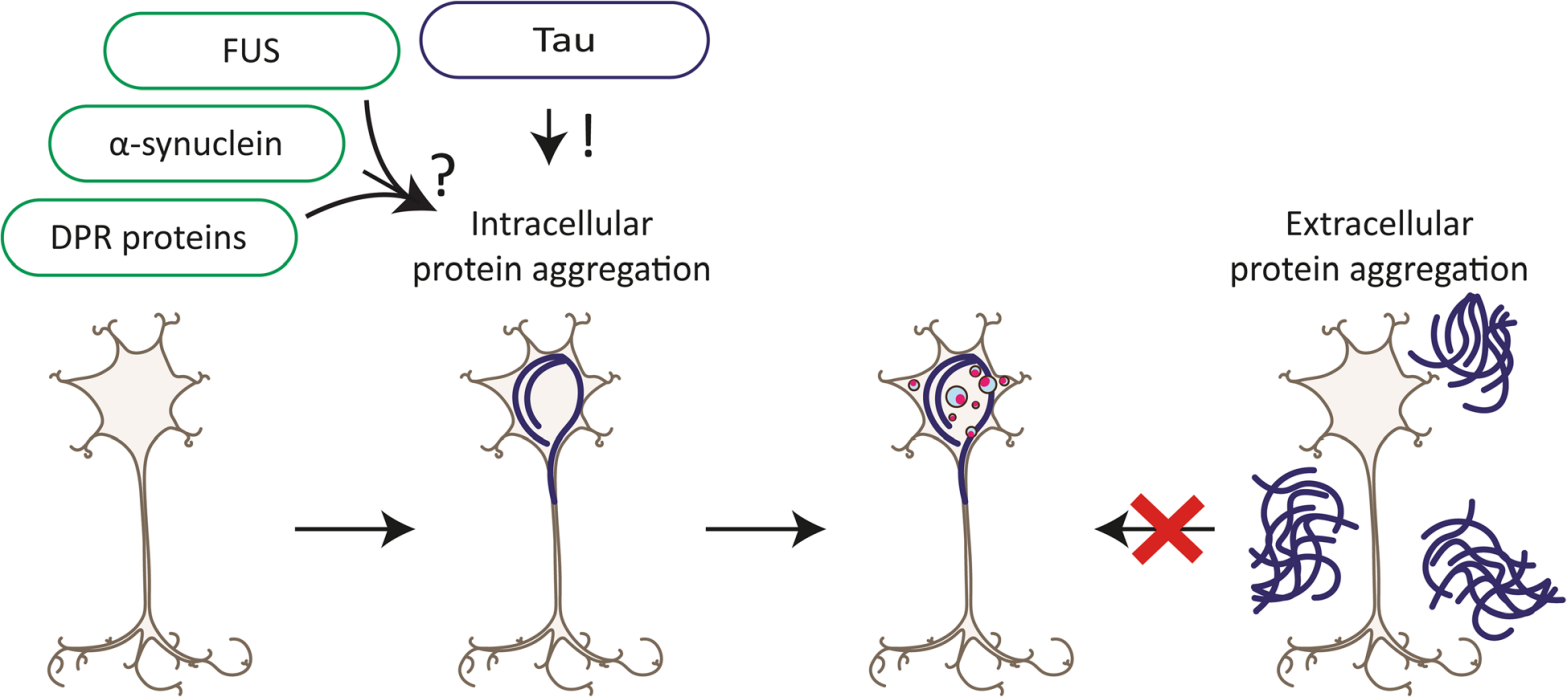
Intracellular protein aggregation causes GVB formation. In the human brain, tau pathology and GVBs co-exist on the regional and cellular level. This is explained by a causal relation between tau pathology and GVB development, as intraneuronal tau pathology induces GVB formation in experimental models. Based on the reevaluation of available neuropathological data in the present review, it is conceivable that aggregates of intracellular proteins other than tau – including DPR proteins, α-synuclein and FUS – can similarly instigate GVB formation. In contrast, data from the human brain indicate that extracellular protein aggregation is not sufficient to induce GVB formation. See text for details; *blue circles with pink core* GVBs; *blue lines* proteinopathy; *!* causal role proven; *?* causal role hypothesized

Recently, we reported that the seeding of tau pathology causes GVB formation in neurons in mouse brain and importantly in cultured primary mouse neurons [143] (Table 2). Seed-induced tau pathology leads to the formation of GVBs that have the protein signature and morphological characteristics of GVBs in the human brain. Using this first in vitro model of GVB formation, causality was demonstrated: the emergence of intracellular tau pathology causes GVB formation (Fig. 2).

As described above, in the AD, PSP and tau Tg mouse brain, GVBs are typically detected in neurons with tau pathology. More specifically, GVB-bearing neurons often show a diffuse staining of tau antibodies recognizing disease-related phospho-epitopes, indicative of an early – so called “pre-tangle” – stage of tau pathology [43, 65, 67, 96, 125, 146]. This indicates a specific association between GVBs and early tau pathology. However, occasionally GVBs are also observed in cells that appear to be free of tau pathology in the human tauopathy [5, 43, 68, 90, 99, 100, 119, 122, 125, 135] and tau Tg mouse [66, 67] brain. Most quantifications range between ~ 2% and ~ 25% of GVB-bearing cells without immunodetection of tau pathology [5, 66, 67, 90, 122, 135]. This incomplete co-localization between GVBs and tau pathology at the single cell level has led to the hypothetical model that the emergence of GVBs and tau pathology are parallel, independent processes in the human brain (see e.g. [48, 60, 68]). Also in the novel experimental GVB models, a minority of GVBs is observed in neurons without apparent tau pathology [143]. Importantly, those GVB-positive, tau-“negative” neurons are only found in the experimental condition in which tau pathology is induced and never in neurons under control conditions. This causal relation between tau pathology and GVB formation suggests that in GVB-bearing cells without detectable tau pathology, early pathological species of tau are present but below the detection limit. Hence, pathological tau species arising early in the aggregation cascade are likely sufficient to induce GVB formation. Furthermore, it is not unlikely that early pathological changes in tau that occur in (distal) neurites rather than the soma are more difficult to detect, potentially resulting in misclassification of a GVB-containing cell as tau-negative. Yet, the tight connection of GVBs with early stages of tau pathology contrasts with the relative absence of GVBs from cells with mature tau aggregates: GVBs are rarely found in neurons with NFTs [32, 43, 60, 65, 90, 96, 146]. Possibly, GVBs disappear when tau aggregation transits to the final stage. Alternatively, tau aggregation may not progress in cells with GVBs (see *GVB formation as a protective or degenerative response?*).

Although GVBs are detected in multiple brain areas in AD, the hippocampus – more specifically, the CA1/CA2/subiculum region – is the predilection site for GVBs as it shows the earliest and most extensive involvement [132]. This regional preference may indicate selective vulnerability of hippocampal neurons to GVB formation. However, in the in vitro GVB model, neurons derived from either the hippocampus or the cortex of mice developed GVBs to a similar extent when challenged with tau pathology [143]. Furthermore, GVBs also formed in neurons cultured from the mouse striatum [143], a region devoid of GVBs in the AD brain [132]*.* Although it could be argued that primary mouse neurons in culture may lack characteristics present in the context of the brain that determine their vulnerability or that species differences play a role, these experimental data argue against a regional preference for GVB development. Rather, they point to tau pathology as the key determinant of the (extent of) GVB formation. Neuropathological observations indicate a strong connection with tau pathology, but also suggest that the extent of GVB formation in tau-positive neurons may display regional differences. In AD, GVBs are absent from the basal ganglia [132], but tau pathology in this brain area is also minor – with moderate NFT load only at the final Braak NFT stage VI [13]. In contrast, both GVBs and tau pathology are found in the basal ganglia of patients with PSP [99, 118, 123]. This suggests that independent of the brain region, neurons form GVBs when tau pathology is present. In agreement, the distribution patterns of tau pathology and GVBs in the AD [12, 13, 132] and tau Tg mouse [67, 90] brain largely overlap. However, in the earliest AD stages, there is an interesting discrepancy as the entorhinal cortex is the predilection site for tau pathology, but the CA1/CA2/subiculum region for GVBs [8, 12, 13, 132]. In addition, in the final AD stages, NFTs are abundant in the frontal, parietal and occipital neocortex, yet GVBs remain scarce in these areas [13, 132] Although precise quantification of the tau and GVB load is needed to verify these observations, it suggests a model where tau pathology is a prerequisite for GVB formation, but additional factors modulate the process and thereby underly selective vulnerability of specific neuronal populations for GVB development. Another possibility is that yet uncharacterized differences in the kinetics or structure of tau pathology between brain regions/neuronal populations dictate GVB formation.

In addition to the frequent co-occurrence of tau pathology and GVBs within the same cells, some studies have indicated that tau and GVBs may also co-localize on the subcellular level, which has been extensively reviewed previously [65]. In summary, the GVB core is immunolabeled by over ten different phospho-specific antibodies recognizing phospho-epitopes on the tau protein. This contrasts with the weak or absent immunopositivity that is often observed using antibodies that bind tau in a phosphorylation-independent manner. Strikingly, in addition to tau, this differential immunoreactivity of GVBs for phosphorylation-dependent versus phosphorylation-independent antibodies is observed for many other proteins (Table 3). Also in the in vitro GVB model, some GVBs were labeled by a phospho-specific tau antibody [143]. However, when the localization of tau was studied using the direct fluorescence of overexpressed tau tagged with a fluorescent protein, neither diffuse nor aggregated tau was detected in GVBs. Furthermore, no fibrillar structures were found inside GVBs by electron microscopy in cultured neurons [143] and in the human AD brain [50, 103]. Together, these data suggest that there is no preferential accumulation of tau (aggregates) in the GVB core, which does not exclude the possible presence of low levels or fragments of tau. The absence of tau enrichment in GVBs is intriguing, as tau pathology drives the formation of these structures. Perhaps soluble tau is more efficiently recruited to tau aggregates than included in GVBs. Other possible explanations include efficient degradation of tau inside GVBs or selective targeting of non-tau cytosolic cargo to GVBs (see *GVB identity*).

**Table 3:** *Box:* GVB immunogenicity

In the neuropathological literature on proteins found in GVBs, a noteworthy discrepancy in GVB immunogenicity is apparent. Typically, phosphorylation-dependent antibodies recognize GVBs, whereas phosphorylation-independent antibodies targeting the same protein do not. A comprehensive list of proteins detected in GVBs till the year 2016 using either phospho-specific or generic antibodies has been published previously [65]. In the novel experimental GVB model in cultured neurons, tau is detected in GVBs using a phosphorylation-dependent antibody, but not using an antibody-independent approach that employs the overexpression of fluorescent protein-tagged tau [143]. Therefore, it is possible that only a protein fragment containing the epitope accumulates in GVBs, which is a plausible explanation in view of the identification of GVBs as proteolytically active compartment (see *GVB identity*). Alternatively, some phosphorylation-dependent antibodies may bind non-specifically to phospho-epitopes that are immunological mimics of their phosphorylated target protein. Recently, it was published that the necroptosis-related proteins RIPK1 and RIPK3 could be detected in GVBs by generic, phosphorylation-independent antibodies only after dephosphorylation of the human brain tissue [68]. This indicates that for some proteins, phosphatase treatment may be necessary to reveal their GVB localization. Therefore, caution is warranted in the interpretation of the functional implications of GVB localization for proteins that have so far solely been detected using phospho-specific antibodies, until the presence of the total protein is confirmed by immunolabeling or using direct fluorescence in experimental models. Even though the functional significance is unclear, phospho-specific antibodies – such as pPERK, peIF2α, pIRE1α and pTDP-43 – consistently identify GVBs in the human and mouse brain and in cultured mouse neurons and are therefore convenient and reliable GVB markers (Table 1). In conclusion, a clear distinction can be made between immunolabeling for the purpose of GVB detection or for the functional interpretation of the immunopositive signal.	

Importantly, in addition to tau inclusions, AD is characterized by the presence of extracellular, plaque-like deposits of aggregated amyloid β (Aβ). However, the distribution of Aβ deposits [12, 13] and GVBs [132] in the AD brain follow a dissimilar pattern. In line with this, no correlation between GVBs and the occurrence of extracellular Aβ aggregates was found in AD patients [68, 135] and non-demented elderly controls [125]. Furthermore, both tau pathology and GVBs are detected in non-demented controls lacking Aβ plaques [131], indicating that GVB formation occurs independently of extracellular Aβ aggregation. Interestingly, intracellular Aβ has been detected inside GVBs [72] (see also *GVB identity*). Furthermore, a higher GVB load is found in bigenic TAPP mice that express both human tau P301L and human mutant amyloid precursor protein (APP) K670M/N671L (APP^Sw^) compared to JNPL3 mice carrying only the human tau P301L transgene [54, 78] (Table 2). This higher GVB frequency is likely related to the exacerbated tau pathology in TAPP mice rather than the comorbid Aβ pathology, as GVBs are absent in mouse models of pure Aβ amyloidosis by overexpression of APP^Sw^ (Tg2576), APP^Sw,Ind^ (J20), APP^Sw/L^/PS1^M146L^ or APP^Sw^/PS1^dE9^ [54, 67, 90] – although the recent detection of granular structures with an immunogenicity profile that partially overlaps with GVBs in APP^Sw^/PS1^dE9^ mice requires further investigation [148]. Taken together, the data indicate that neither in human nor in Tg mouse brain, GVBs and extracellular Aβ pathology are associated. The conclusion that tau rather than Aβ pathology is the driver of GVB formation in AD is in accordance with the presence of GVBs in many primary tauopathies.

## GVB formation in “non-tau” neurodegenerative proteinopathies

Protein aggregation is a common neuropathological characteristic of many neurodegenerative diseases. Although different proteins form the basis of aggregates in these neurodegenerative proteinopathies, the biochemical mechanisms underlying their formation appear to overlap. Therefore, it is conceivable that aggregating proteins other than tau can also instigate GVB formation.

In addition to tauopathies, the presence of GVBs has been studied in the brains of patients with other neurodegenerative proteinopathies, including human prion diseases, α-synucleinopathies and TDP-43- and FUS-related FTLD and amyotrophic lateral sclerosis (ALS). Importantly, even in the brains of patients with these “non-tau” neurodegenerative diseases that are primarily characterized by the aggregation of another protein, concurrent tau pathology can be observed. This tau pathology is often found in the hippocampal region, probably reflecting aging-/AD-related tau pathology. However, comorbid tau pathology can also be present in disease-specific brain areas, for example in human prion disease patients [70, 112, 142]. Given the causal relation between tau pathology and GVB formation, it is essential to investigate tau co-pathology in patients with “non-tau” neurodegenerative proteinopathies. Only upon exclusion of tau pathology as a confounding factor, a conclusion on the correlation between other protein aggregates and GVBs can be drawn. Therefore, in Table 4, the probability of tau co-pathology is reviewed for each of the studies discussed below. In this assessment, we have also made use of the fact that phospho-specific antibodies targeting tau and TDP-43 (see *GVBs as pathological companion of tau pathology*; *GVB identity*; Table 3) recognize both protein aggregates and GVBs (see e.g. [47, 48, 59]). Accordingly, if p-tau or pTDP-43 staining labels GVBs but not aggregates, this is an indication that tau/TDP-43 protein pathology is absent from those cells. It should be mentioned that detection is dependent on antibody titration and image acquiring settings that may vary between different studies. In addition, the detection of low abundant pathological protein species by qualitative microscopy is a general limitation. Lastly, it is important to note that the extent of evidence of a GVB identity varies between the studies discussed below. To streamline future studies, we have formulated three criteria for the assessment of GVB identity (Table 1; Fig. 1 ; see *Recommendations and concluding remarks*). In Table 4, for each study the evidence to confirm a GVB identity is scored based on the novel criteria. Below, neuropathological data on putative GVBs in “non-tau” neurodegenerative proteinopathies is discussed.

**Table 4:**
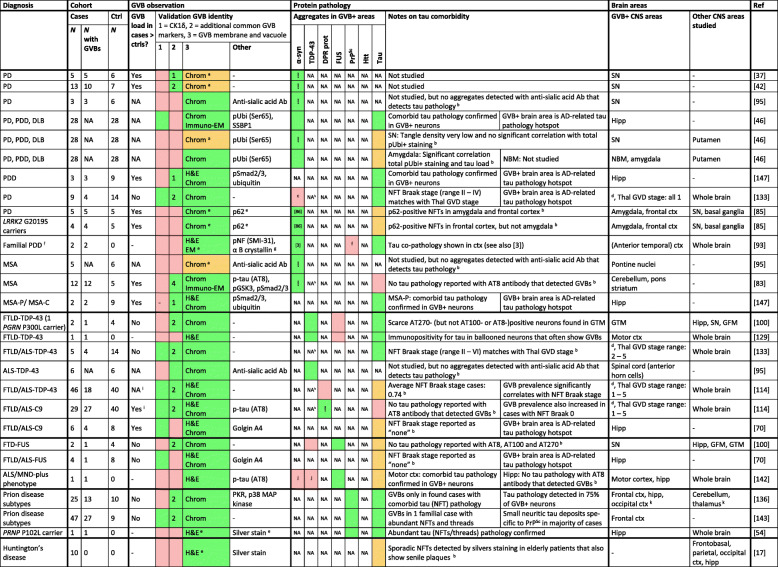
Putative GVB observations and comorbid tau pathology in “non-tau” neurodegenerative proteinopathies

Diagnosis: clinical diagnosis and if applicable causative mutations are listed. Cohort: the number (*N*) of cases, of cases in which GVBs are detected and of non-neurological controls are listed. GVB observation: if a control group was included, it is indicated whether the GVB load was higher in cases than in controls. Color-coding is used to denote if studies meet the criteria for validation of GVB identity (Table 1; Fig. 1). *Green*: criterion met; *red*: criterion not met; *orange*: see ^*a*^. For criterion 2, the number of additional common GVB markers used in the study is noted. For criterion 3, the method used is indicated. Other antibodies and methods used to probe GVBs are listed, with antibody names or phospho-epitopes shown in brackets. Protein pathology: Color-coding is used to denote the presence of protein aggregates in those brain areas where GVBs were detected. *Green*: detected (reference to publications showing the presence of these protein aggregates is included if it was not shown in the same study); *red*: not detected; *orange*: possible tau comorbidity, see additional notes hereon in the next column; *NA*: not available (either this protein was not studied or no data is available on the presence of this protein specifically in the GVB-positive brain areas); *!* co-localization of non-tau protein aggregates and GVBs in the same cells. Additional notes on the detection of tau pathology in GVB-containing brain areas are listed. For NFT Braak staging see [13]. CNS areas: CNS areas where GVBs were and were not reported are listed. Reference to publications are included (Ref). ^*a*^ In these studies, GVB-like structures were described as smaller granules/punctate structures as the GVB vacuole and membrane did not stand out upon chromogenic detection. Notably, other chromogens than DAB were used given the presence of neuromelanin in studied neurons. ^*b*^ Comorbid tau pathology was not conclusively excluded in GVB-containing cells. ^*c*^ Probably absent, based on a PD Braak stage of 4-5. For PD Braak staging see [14]. ^*d*^ Thal GVD staging is performed and the range of Thal GVD stages is reported for those cases with GVBs. For brain areas affected by GVBs in each Thal GVD stage see [132]. ^*e*^ No typical example of putative GVBs shown (in case of Huntington’s disease because no GVBs were found). ^*f*^ A later report stated that this was probably the *SNCA* G51D mutation [62]/PrP^Sc^ pathology was excluded in 1 of 2 cases. ^*g*^ Putative GVBs were visible due to strong staining of the whole cytoplasm rather than GVB-specific staining. ^*h*^ pTDP-43 antibody was used to detect GVBs and no pTDP-43-positive inclusions were reported in GVB-bearing cells. ^*i*^ FTLD/ALS-C9, but not FTLD/ALS-nonC9, cases were directly compared to controls. ^*j*^ Comorbid α-synuclein and TDP-43 pathology excluded in motor cortex, but not hippocampus. ^*k*^ Occipital cortex, cerebellum and thalamus were only studied for specific prion disease subtypes. A single positive neuron was detected in the occipital cortex of one case. Note that for clarity Aβ comorbidity is not included in this table

Abbreviations: *AD* Alzheimer’s disease, *ALS* amyotrophic lateral sclerosis, *Chrom* GVB morphology visualized by chromogenic peroxidase-catalyzed immunodetection of GVB core marker, *CNS* central nervous system, *Ctrl* controls, *Ctx* cortex, *DLB* Dementia with Lewy bodies, *DPR prot* dipeptide repeat proteins, *FTLD* frontotemporal lobar degeneration, *FTLD/ALS-C9* TDP-43 related FTLD/ALS caused by a hexanucleotide repeat expansion in C9ORF72, *FTLD-FUS* FUS-related FTLD, *FTLD/ALS-FUS* FUS-related FTLD/ALS, *FTLD-TDP-43* TDP-43-related FTLD, *FTLD/ALS-TDP-43* TDP-43-related FTLD/ALS, *GFM* gyrus frontalis medialis, *GTM* gyrus temporalis medialis, *GVB* granulovacuolar degeneration body, *GVB+* GVB-positive/-containing, *GVD* granulovacuolar degeneration, *H&E* hematoxylin and eosin, *Hipp* Hippocampus, *Immuno-EM* electron microscopy with immunodetection of GVB marker, *LB* Lewy bodies, *MND* motor neuron disease, *MSA* multiple system atrophy, *MSA-C* MSA with cerebellar ataxia, *MSA-P* MSA with parkinsonism, *NBM* nucleus basalis of Meynert, *NFT* neurofibrillary tangle, *p* phosphorylated, *PD* Parkinson’s disease, *PDD* Parkinson’s disease with dementia / parkinsonism and dementia, *Ref* reference, *SN* substantia nigra, *>* higher than, *-* not applicable. For abbreviations of protein and gene names and antibodies see “List of abbreviations”

The pathological prion protein (PrP^Sc^) predominantly aggregates extracellularly in the brains of patients with human prion diseases. For PrP^Sc^ deposits, no association with GVBs was found by two independent studies covering the majority of human prion disease subtypes [135, 142] (Table 4). Interestingly, in one of these cohorts [142], almost all prion disease cases displayed small neuritic tau-positive inclusions that are believed to be secondary to PrP^Sc^ deposition and are morphologically different from AD-type tau pathology [70, 112]. GVBs were hardly observed in these prion disease patients, indicating that modest, distal PrP^Sc^-related changes in tau are not associated with GVB formation. On the other hand, abundant GVBs were observed in a prion disease patient in which a *PRNP* mutation led to an unusual Gerstmann-Sträussler-Scheinker (GSS) clinical phenotype [142]. In this patient, PrP^Sc^ deposition was accompanied by severe tau pathology – including numerous tangle-like structures and neuropil threads – whereas Aβ pathology was absent [57, 142]. Similarly, GVBs have been described in another familial GSS patient with pre-tangles and NFTs [53]. Furthermore, GVBs were found in the hippocampus of prion disease patients with comorbid AD-related tau pathology [135]. GVBs are therefore specifically detected in prion disease patients with proximal tau pathology in the form of NFTs, suggesting that in these cases the tau pathology is the cause of GVB formation. As neither extracellular deposits of aggregated PrP^Sc^ nor of Aβ correlate with the presence of GVBs in the human brain, it appears that extracellular protein aggregation does not trigger GVB formation (Fig. 2).

Post-mortem studies have also investigated the presence of GVBs in neurodegenerative proteinopathies characterized by the intracellular aggregation of proteins other than tau, albeit generally in small cohorts (Table 4). In a single study, GVBs were not found in different brain regions of 10 patients with Huntington’s disease [17]. In contrast to the tau-related FTLD variants, no association was found between GVBs and protein aggregates composed of FUS in different brains areas of patients with FUS-related FTLD [99]. In another study, scarce GVBs were found in the hippocampus of only 1 out of 4 patients with FUS-related FTLD/ALS [69]. Interestingly, in a case report, GVBs were found in the motor cortex and hippocampus of a patient with an ALS-like phenotype and FUS inclusions [141]. The presence of GVBs in the motor cortex of this case may be explained by the detection of comorbid tau pathology in the same motor neurons. However, in the hippocampus, the p-tau antibody AT8 labeled GVBs, but in the same cells tau aggregates were not reported [141]. These data from a single patient may point to tau-independent GVB induction by FUS aggregates.

GVBs are not associated with aggregates of TDP-43 in different brain areas of patients with TDP-43-related FTLD [99] and also do not localize to neurons with TDP-43 inclusions in AD patients with comorbid TDP-43 pathology [59]. Furthermore, neither TDP-43 aggregates nor nuclear clearance of TDP-43 – a cellular change that is often associated with cytoplasmic TDP-43 aggregation – were observed in neurons with GVBs in a large cohort of elderly brain donors [47]. Also in anterior horn cells with TDP-43 pathology in the spinal cord of ALS patients, no GVBs were detected [94]. These data indicate that there is no association between TDP-43 aggregates and the presence of GVBs. In line with this, in two studies that did describe GVBs in patients with TDP-43 proteinopathy [128, 132], GVB detection was likely related to comorbid tau pathology. In the first study, GVBs were found in the hippocampus of TDP-43 proteinopathy patients with NFT Braak stages ranging between II and VI, but not in a TDP-43 case with NFT Braak stage 0 [132]. Furthermore, GVBs in this study were detected using an antibody against pTDP-43 that recognizes both aggregates and GVBs, but in neurons with pTDP-43 immunoreactive GVBs, no protein inclusions were reported, suggesting that there is no robust TDP-43 aggregation in those cells. In the second study, GVBs were found in ballooned neurons in the motor cortex of an FTLD/ALS patient with TDP-43 inclusions [128]. However, also tau pathology was often detected in ballooned neurons and may explain the appearance of GVBs. Together, these data do not support an association between TDP-43 aggregation and GVB formation.

Interestingly, GVBs were found to be more prevalent and more widely distributed in patients with TDP-43-related FTLD/ALS carrying a hexanucleotide repeat expansion in *C9ORF72* (FTLD/ALS-C9) compared to TDP-43-related FTLD/ALS patients without a *C9ORF72* repeat expansion and age-matched controls in an extensive study [113]. In line with this, low levels of GVBs were also reported in the hippocampus of 4 out of 6 FTLD/ALS-C9 patients in another study [69]. In addition to TDP-43 inclusions, FTLD/ALS-C9 patient brains contain aggregates composed of dipeptide repeat (DPR) proteins that result from non-canonical translation of the expanded repeat region. In FTLD/ALS-C9 patients, GVBs were frequently found in those neurons that also contained DPR inclusions [113]. On the other hand, using a pTDP-43 or p-tau antibody in the same patients, GVBs were clearly detected, yet no aggregates were visible in the same neurons. These observations strengthen the idea that DPR protein rather than TDP-43 or tau aggregates are associated with the presence of GVBs in this cohort. Furthermore, the GVB prevalence in FTLD/ALS-C9 cases was also increased when only subjects with the NFT Braak stage 0 were taken into account [113]. Therefore, the presence of GVBs seems specifically related to the presence of DPR protein aggregates and not to concomitant tau pathology in these FTLD/ALS-C9 patients.

Additional studies have reported the presence of (putative) GVBs in brain areas and cells with α-synuclein aggregates in patients with multiple system atrophy (MSA) [82, 94, 146] and patients in the Lewy body disease spectrum, including Parkinson’s disease [37, 42, 45, 84, 94, 132] and Parkinson’s disease with dementia [45, 92, 146], with both sporadic and genetic etiologies. Interestingly, GVBs are frequently found in cells with early stages of α-synuclein aggregation in MSA patients [82], mirroring the specific association of GVBs with early tau pathology (see *GVBs as pathological companion of tau pathology*). In some of the α-synucleinopathy subjects, tau comorbidity was detected either at the single cell level in GVB-carrying neurons [45, 146] or in the same brain areas as the GVBs, as shown by the additional immunodetection of pathological tau [92] or based on the NFT Braak staging of the cases [132]. Yet, concurrent tau pathology does not seem to fully explain the detection of GVBs in all α-synucleinopathy cases. In MSA patients, although the GVBs were labeled by the p-tau antibody AT8, no tau pathology was noted in GVB-positive neurons [82]. Furthermore, GVBs were found in brain areas specifically affected by α-synuclein pathology in MSA patients, namely cerebellum, pons and striatum [82]. In line with this, GVBs – and smaller granules – were detected in α-synuclein aggregate-bearing neurons in the substantia nigra of three patients with Parkinson’s disease [94]. As this brain area becomes mildly affected by AD-related tau pathology only at NFT Braak stage V [13], AD-related tau co-pathology in all cases seems improbable. However, the presence of comorbid tau pathology cannot be fully excluded without analysis at the single cell level. In addition, several independent studies have reported granular structures possibly representing (small) GVBs in cells with α-synuclein pathology in disease-specific brain areas, namely in the substantia nigra of Parkinson’s disease [37, 42] and Lewy body disease spectrum [45] patients and in the pontine nuclei of MSA patients [94]. The GVB identity of these granules remains to be validated.

Taken together – and taking into consideration incomplete evidence for GVB identity and tau pathology as confounding factor (Table 4) – the limited neuropathological data available suggest that, in addition to tau, also α-synuclein, DPR protein and possibly FUS aggregates may be able to elicit GVB formation in the human brain (Fig. 2). GVB formation may thus be a common response to intracellular protein aggregation.

## GVB identity

Detailing all proteins detected in GVBs is beyond the scope of this review and for an overview of proteins detected in GVBs up to 2016 the reader is here referred to a previous, excellent review [65] (for novel GVB-localizing proteins see below). Here, the molecular composition of GVBs is briefly discussed in the light of recent discoveries in the human brain and experimental models. In Table 1, common markers of GVBs in the human brain and experimental models and the level of rigor to which these markers have been validated are discussed and in Supplementary Table 1, primary antibodies for common GVB marker detection are listed. For other proteins localizing to GVBs discussed below, the extent of evidence confirming their GVB localization (e.g. the number of publications and the study of overlap with common GVB markers) varies. Investigating the molecular composition of GVBs is important, as it provides clues to the mechanism of their formation.

GVBs were previously proposed to be an aberrant type of autophagosome: a double-membraned autophagic intermediate [103]. This hypothetical GVB origin has been widely referred to and over time turned into a definition of GVBs. However, neither GVBs in the human brain [30] nor experimental GVBs [143] are immunopositive for the autophagosome membrane marker LC3. This is in agreement with ultrastructural analysis using immunoelectron microscopy in both post-mortem tissue [33, 74] and the in vitro GVB model [143] that did not show a double membrane, as would be expected if GVBs were autophagosomes. In agreement with the presence of a single limiting membrane, the GVB membrane is positive for the lysosomal transmembrane proteins LAMP1 [30, 143] (Fig. 1f) and LIMP2 [143]. Furthermore, the lysosomal hydrolase CTSD is found in GVBs in the human brain [30, 94] and the in vitro GVB model [143]. Using the endocytic cargo and cathepsin substrate DQ-BSA – of which the fluorescence becomes dequenched upon proteolysis – it was shown that indeed many GVBs in vitro are proteolytically active [143]. Quantification of the DQ-BSA fluorescence intensity revealed that the proteolytic activity in GVBs is similar to that of other degradative compartments in the same neuron. In the human brain, GVBs showed stronger co-localization with LAMP1 than with CTSD [30]. This is in line with data from the in vitro GVB model that consistently shows a LAMP1/LIMP2-positive GVB membrane, but a range of CTSD and DQ-BSA intensity values in the population of GVBs [143], which could indicate variation in the degradative capacity between individual GVBs. The predominant somatic localization of GVBs mimics the subcellular distribution of proteolytically active lysosomes in neurons that are more abundant in the soma than the neuronal protrusions in contrast to earlier organelles in the endo- and autolysosomal pathways [28, 150]. In conclusion, combined post-mortem and experimental data showing a single limiting membrane, the presence of lysosomal membrane and proteolytic proteins as well as degradative capacity therefore identify GVBs as active lysosomal structures, rather than autophagosomes. GVBs can be distinguished from physiological lysosomal structures by the accumulation of a dense protein core, despite their proteolytic activity. The accumulation of endocytic and cytosolic cargo in GVBs indicates that both endolysosomal and autolysosomal pathways contribute to their content.

The presence of the endocytic cargo DQ-BSA in GVBs [143] shows that extracellular content can reach the degradative GVB lumen via the endolysosomal pathway. In line with this, various other markers along the endolysosomal pathway localize to the GVB core. Studies have identified multiple markers of late endosomes in GVBs, including Rab7 and M6PR in GVBs in the human brain [148] and CHMP2B in GVBs in the human and mouse brain and cultured cells [30, 90, 143, 147]. Also the CHMP2B-interacting protein VPS4a has – at low levels – been found in GVBs [90]. Some studies additionally identified the early and recycling endosome marker phosphorylated Rab10 (Table 3) [149], the recycling endosome marker Rab11 [148] and the early endosome marker EEA1 [148] in human GVBs. However, in the GVB model in cultured neurons EEA1 was absent from GVBs – whereas early endosomes were clearly detected in a punctate staining pattern –, indicating a more prominent involvement of late than early stage endosomal proteins in GVBs. In line with this, also the membrane-associated protein Flotillin-1 that localizes to the plasma membrane as well as the membrane of late endosomes and lysosomes, co-localizes with GVBs in the human and mouse brain [95, 148]. Flotillin-1 is additionally being used as an exosomal membrane marker, raising the possibility that some GVBs undergo exocytosis, although GVBs are typically not observed in association with the plasma membrane and to date experimental data supporting GVB release is lacking.

A variety of proteins accumulates in the characteristic GVB core. Human GVBs have been reported to contain ubiquitin [25, 45, 80, 103] and the autophagy receptor p62 [59, 84], although this is not consistently observed (ubiquitin: [25, 43, 80, 103]; p62: [30, 43, 113]). This indicates a connection with disturbed proteostasis, which is further strengthened by the presence of protein factors involved in proteostatic stress responses in the GVB core. Human and experimental GVBs are immunopositive for the phosphorylated forms of key proteins in the unfolded protein response (UPR), namely pIRE1α, pPERK (Fig. 1c-f) and its downstream target peIF2α [43, 67, 143]. The phosphorylated state of these proteins is indicative of activation of the UPR: a cellular stress response initiated upon disturbances of the protein folding homeostasis in the endoplasmic reticulum (ER; Table 3). Also the UPR-induced ER-resident E3 ligase Hrd1 is present in GVBs [44]. In addition to UPR activation markers, other cellular stress-related proteins have been found in GVBs in the human brain, including key mediators in apoptotic signaling cascades, such as caspase-3 [122, 126] and phosphorylated SAPK/JNK [74], and the phosphorylated/activated necrosome complex proteins RIPK1, RIPK3 and MLKL (Table 3, the presence of total RIPK and RIPK3 in GVBs was confirmed after tissue dephosphorylation [68]). Furthermore, disease-associated amyloidogenic proteins are detected in GVBs in post-mortem tissue. Despite the lack of association of extracellular Aβ plaque pathology with GVB occurrence, both phosphorylated and non-phosphorylated Aβ have been detected in the GVB core [72]. As discussed above, data from the in vitro GVB model indicate that tau does not accumulate in GVBs albeit their frequent immunopositivity for phospho-specific anti-tau antibodies (see *GVBs as pathological companion of tau pathology*; Table 3). Similarly, TDP-43 has been found in GVBs in the human and mouse brain using phosphorylation-dependent [47, 59, 79, 148], but not generic anti-TDP-43 antibodies [47, 59, 79] (Table 3). Furthermore, FUS immunoreactivity was detected in GVBs in the human – but not tau Tg mouse – brain [148]. Also various kinases that can contribute to pathological hyperphosphorylation of tau are found in GVBs in the human brain, often in phosphorylated/active form (Table 3). Tau kinases that have been reported to localize in GVBs include GSK-3β [43, 76], CDK5 [96], MARK3 and MARK4 [81], SAPK/JNK [74], PSKs [129], p38 MAP kinase [153], Syk [116], c-Abl [58] and CK1δ [33]. In addition to CK1δ (Fig. 1a, b), also the casein kinase 1 isoforms CK1α and CK1ε have been detected in human [33] and human and experimental GVBs [33, 67, 143], respectively. Interestingly, in the in vitro GVB model it was shown that green fluorescent protein (GFP)-tagged CK1δ but not GFP-tau or GFP alone accumulates in GVBs [143]. This suggest that selective targeting or degradation mechanisms are at play in GVBs. Furthermore, a plethora of other proteins involved in various cellular processes have been found in GVBs (for a tabular overview of GVB-localizing proteins discovered till 2016 see [65]; in our literature search, we additionally came across publications reporting GVB localization in tissue for PKR [135], phosphorylated p300 [5], PICALM [2], annexin2 [95], LRRK2 [95] and reticulon-3 [36] and publications after 2016 showed the GVB localization of TMEM230 [119], Dvl3 [93], rapsyn [93], APC [93], PrP [142], Golgin A4 [69], phosphorylated (S65) ubiquitin [45], SSBP1 [45], nucleolin [38], SIL1 [73], NF-κB [145], GM130 [148], β-COP [148], matrin-3 [148], G3BP [148] and immunoreactivity of GVBs for an anti-sialic acid antibody [94] (for phospho-specific antibodies see Table 3)). In conclusion, regardless of the presence of proteolytic activity markers, the GVB core harbors a variety of proteins, including various markers of cellular stress and potentially harmful proteins.

## Untangling the mechanism: GVB formation as a lysosomal stress response to intracellular protein aggregation?

GVBs are lysosomal structures that form in response to (early) aggregates of tau and possibly other proteins. In search of the mechanism underlying protein aggregation-induced GVB formation, the cell type-specific appearance of GVBs is of interest. In the human brain, GVBs have predominantly been reported in neurons. Neuropathological reports on GVBs in glia cells are scarce, but GVBs have been reported in glia with tau pathology in patients with Pick’s disease and FTLD caused by *MAPT* mutations [99] and in oligodendrocytes with α-synuclein pathology – the so-called “glial cytoplasmic inclusions” or “Papp-Lantos bodies” – in MSA patients [82]. Contradictory data have been reported on the presence of GVBs in glia with tau pathology in patients with PSP or a mixed PSP/CBD phenotype [99, 118, 135]. GVBs were not detected in glia in patients with the tauopathies aging-related tau astrogliopathy [90], PPND and parkinsonism dementia complex of Guam [118]. Taken together, the much larger body of literature on GVBs in neurons relative to that on GVBs in glia in the human brain suggests a neuron-selective, but not neuron-exclusive, occurrence of GVBs. Experimental GVB formation is also a neuron-selective process: GVBs form in cultured primary neurons, but not primary astrocytes or HEK293 cells with seeded tau pathology [143]. These data suggest that the neuronal predominance of GVBs in the human brain is caused by a cell type-specific response to tau pathology rather than by a different susceptibility of neurons and glia to develop tau pathology. This indicates that GVBs are formed via a mechanism that is more readily induced by intracellular protein aggregation in neurons than in glia.

**Fig. 3:**
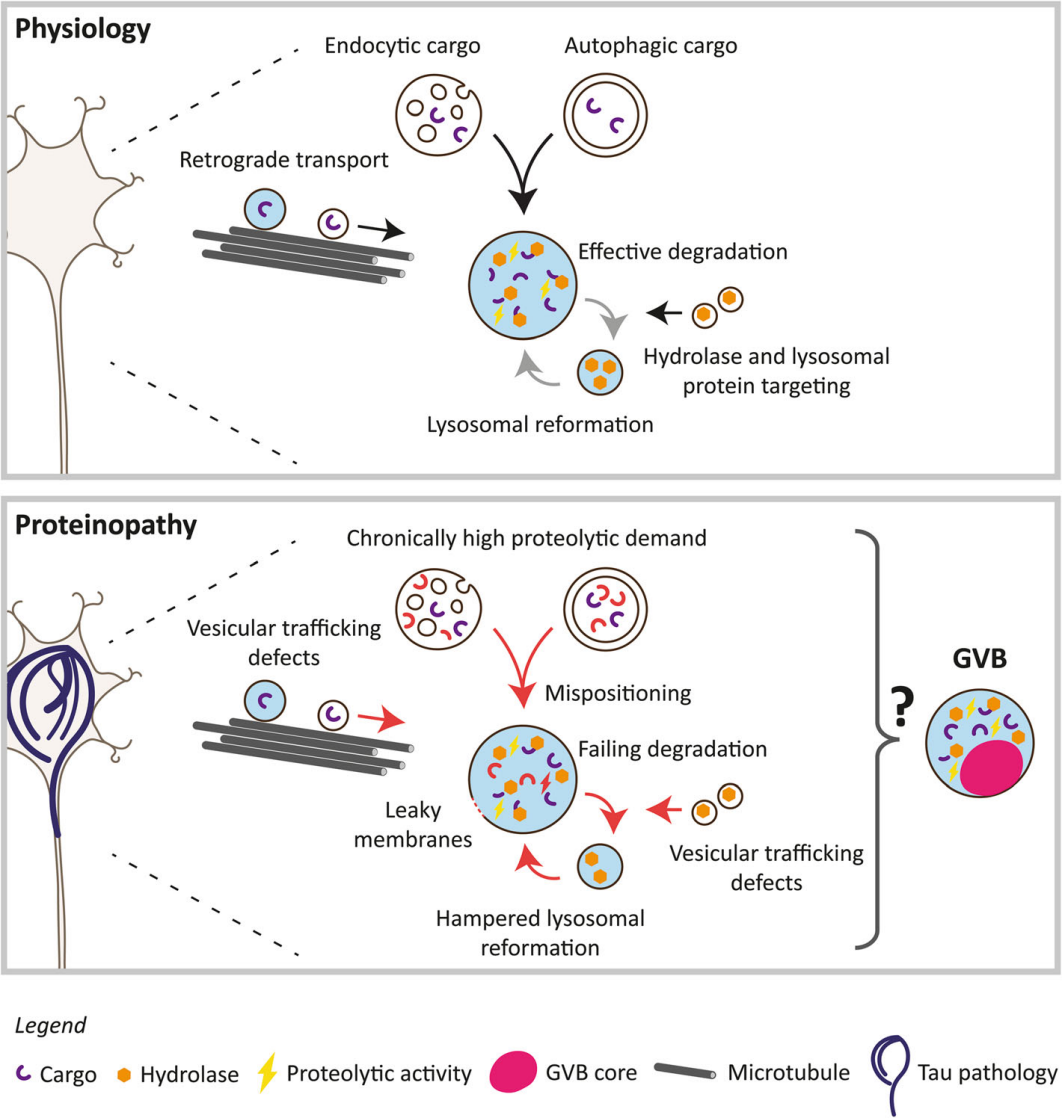
Hypothetical mechanisms involved in GVB formation. Under physiological conditions, endocytic and autophagic cargo is transported to the soma, where it is degraded in lysosomes. Efficient lysosomal degradation is safeguarded by the lysosomal reformation cycle and the delivery of essential lysosomal proteins and hydrolases. Proteinopathy, such as tau pathology, leads to the formation of GVBs that have been identified as lysosomal structures based on the presence of a single limiting membrane, lysosomal transmembrane proteins, the hydrolase CTSD and a proteolytic activity marker. Yet, GVBs harbor a characteristic dense proteinaceous core distinguishing them from physiological lysosomes. This indicates that protein aggregation alters the lysosomal system in such a way that the GVB-type lysosomes are formed. Possible protein aggregation-induced mechanisms that may contribute to GVB formation are shown. See text for details

The identification of GVBs as lysosomal structures indicates that intracellular protein aggregation changes the lysosomal system in such a way that the GVB-type lysosomal structures are formed (Fig. 3). Disruptions in the lysosomal system have been found in the human brain in close connection to the aggregation of intracellular proteins including tau [101, 102, 107, 140] and α- synuclein [20, 23]. Evidence from cell models suggests that this may be a direct functional consequence of protein aggregation. For example, studies in human induced pluripotent stem cell (iPSC)-derived neurons have shown reduced lysosomal acidification upon the expression of mutant P301S tau [137] and decreased lysosomal proteolysis upon the accumulation of α-synuclein [89]*.* Disturbance of the lysosomal system as the mechanistic link between protein aggregation and GVB formation fits with the differential susceptibility of neurons and glia cells for GVB formation. In contrast to glia cells that retain at least some regenerative capacity by proliferation, post-mitotic neurons are highly dependent on their lysosomal system for survival. Additionally, the high polarity of neurons requires long-distance trafficking of (lysosomal) organelles and cargo. Therefore, neurons are likely to respond differently to (protein aggregation-induced) lysosomal stress than glia cells. This is illustrated by the finding that neurodegeneration is a prominent feature of lysosomal storage disorders (LSDs), which are caused by mutations in genes encoding ubiquitously expressed lysosomal enzymes and other lysosomal proteins [104]. Interestingly, case studies have reported the presence of GVBs in patients with the LSDs Niemann-Pick disease type C [127] and Salla disease [6]. In these patients, also NFTs were detected in the GVB-containing brain areas. Therefore, although further study of the LSD spectrum is warranted, this could indicate that also in these disorders concomitant protein aggregation is a prerequisite for the development of GVBs.

As discussed above, the aggregation of proteins other than tau may similarly elicit GVB formation. Consistent with this hypothesis, GVB formation could not be directly connected to loss of the physiological function of tau, as acute disruption of the microtubule network in cultured primary neurons does not lead to GVB formation [143]. Therefore, a generic gain-of-function response of the lysosomal system to (early) intracellular protein aggregates appears to underlie the formation of GVBs (Fig. 3). Protein aggregates and cellular components damaged by those aggregates will be targeted for degradation, demanding an increase in the degradative capacity of the lysosomal system. Persistent protein aggregation may overload the lysosomal system and the protein accumulation in the GVB core may therefore represent an overwhelmed lysosome. This is in line with the possible detection of different amyloidogenic proteins and ubiquitin in GVBs. In addition to a direct overload of lysosomal proteolysis, protein aggregates may disrupt the integrity of the lysosomal membrane, resulting in leakage of lysosomal enzymes to the cytosol. Indeed, in tauopathy patients the lysosomal hydrolase CTSD shows a diffuse cytoplasmic localization, whereas in controls CTSD is observed in punctate structures corresponding to lysosomes [107]. It has been shown that tau aggregates impair the membrane integrity of artificial phospholipid vesicles [29] and that extracellularly supplied tau aggregates have the ability to damage the membrane of endocytic vesicles in cultured rat neurons [19], indicating that pathological forms of tau can rupture vesicular membranes. Lysosomal membrane permeabilization likely causes a drop in proteolytic capacity. In turn, this may interfere with the process of lysosomal reformation, as efficient degradation of auto- and endolysosomal cargo is required for regeneration of the lysosomal pool [152]. Retrograde axonal transport is essential for the maturation of lysosomes and thereby proper cargo degradation in neurons [27]. Another possibility therefore is that protein aggregates indirectly induce lysosomal stress via the disruption of vesicular transport. Indeed, reduced axonal transport of lysosomes is observed in cultured mouse neurons with tau aggregates [35]. Also α-synuclein aggregates disrupt vesicular protein trafficking, including the targeting of hydrolases to lysosomes early in the secretory pathway, leading to lysosomal dysfunction in human iPSC neurons [89]. Therefore, protein aggregation-induced trafficking defects can lead to mispositioning of lysosomes and impaired cargo delivery. Protein aggregation therefore not only imposes a chronically high proteolytic demand on a neuron, but may also lead to failing degradation, hampered lysosomal reformation and defective trafficking of lysosomes and their cargo. Any of these processes alone or in combination leads to a situation of stress in the lysosomal system that may in turn result in the appearance of the GVB-type lysosomal structure (Fig. 3).

## GVB formation as a protective or degenerative response?

**Fig. 4:**
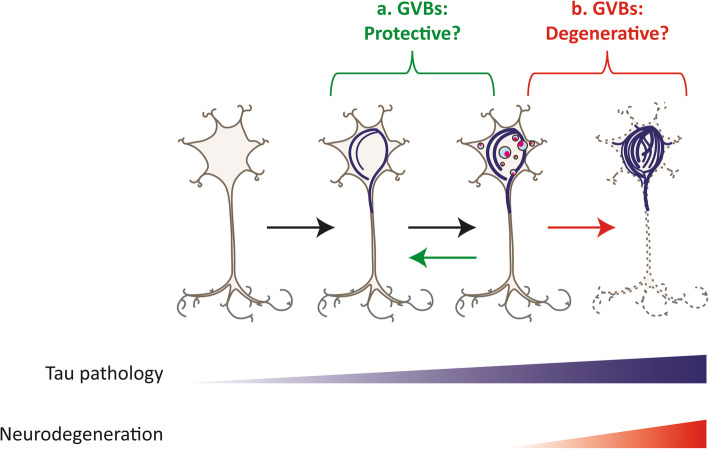
GVB formation as a protective or degenerative response to proteinopathy? The intracellular aggregation of tau and possibly other proteins elicits GVB formation, a response that may be either **a** protective or **b** degenerative. **a** GVBs could be protective by boosting the lysosomal system, increasing the cells proteolytic capacity or isolating harmful proteins from the cytosol, thereby obviating or reversing ongoing pathological proteinopathy and neurodegeneration. As such, proteinopathy may not proceed to the end-stage in GVB-bearing neurons. **b** Alternatively, GVBs could signify collapse of the lysosomal system, indicating that the cell is beyond rescue and that proteinopathy will irreversibly progress to the end-stage, kicking-off neurodegeneration. Note that GVBs are not drawn in the degenerating neuron, which is in line with the absence of GVBs from neurons with NFTs. See text for arguments in favor of both hypothetical functional outcomes of GVB formation; *blue circles with pink core* GVBs; *blue lines* tau pathology/proteinopathy; *dashed neuron* neurodegeneration

An outstanding question is whether GVB formation is a protective or degenerative response to intracellular protein aggregation (Fig. 4). GVBs predominate in cells with early-stage protein aggregation, as shown for tau [43, 65, 67, 96, 125, 146] and α-synuclein [82]. The emergence of GVBs early in the protein aggregation process could indicate a protective response, aimed to make neurons more resilient to proteostatic stress. Furthermore, the active contribution of GVBs to the degradation of cellular waste suggests that GVBs are protective structures, formed to restore proteostasis by increasing the degradative capacity of the neuron. Lysosomal stress can trigger the transcription factor EB (TFEB) response, which activates a transcriptional program that promotes cellular clearance by enhancing lysosomal biogenesis and autolysosomal flux [21]. TFEB overexpression reduces the tau pathology load in tau Tg mice [108, 139]. This demonstrates that lysosomal stress responses can have neuroprotective consequences in the context of protein aggregation. The GVB-type lysosomal response may have a similar beneficial outcome. In favor of a protective role, highly abundant GVBs were reported in a cohort of cognitively healthy individuals over 100-years-of-age [31]. This may suggest that in healthy centenarians, a high GVB load is associated with protection from neurodegeneration and clinical symptoms. In line with this, phosphorylated/activated stress signaling proteins involved in adaptive responses like UPR activation are found in GVBs, which could indicate that a protective response is ongoing. Indeed, different downstream targets of the UPR signaling cascades are upregulated in brains of patients with neurodegenerative diseases [117]. Although these observations are in agreement with UPR activation in GVB-positive neurons, a functional involvement of the UPR in GVB formation has not been demonstrated (Table 3).

Alternatively, the presence of GVBs in neurons with early-stage protein aggregation [43, 65, 67, 82, 96, 125, 146] may indicate defects in the lysosomal system due to the persistent proteostatic stress. The presence of the dense proteinaceous core in GVBs, despite their proteolytic activity, could reflect failure to keep up with the proteolytic demand of the cell under proteostatic stress. Hence, the presence of GVBs may indicate a transition towards full-blown protein aggregation and cell death. In line with this, post-mortem studies have shown that neurons with GVBs display nuclear and nucleolar damage [87, 126] – indicative of apoptotic processes – yet these findings have been contradicted by others [122, 130]. Different proteins involved in cell death pathways have been identified in GVBs, often in their active form, including pro-apoptotic signalers, pro-necroptosis factors, amyloidogenic proteins and tau kinases (Table 3). The presence of these potentially toxic proteins inside GVBs has been interpreted in different ways. Possibly, it indicates a protective mechanism: GVBs may sequester these proteins from the cytosol to obviate their harmful actions by spatial separation, thereby delaying further (tau) pathology or other cellular damage. For example, the presence of the activated necroptotic complex in the GVB core rather than at the plasma membrane [68] – where it can execute its function in cell death – may indicate shielding of these proteins inside GVBs as a protective mechanism. Alternatively, the presence of pro-necroptotic and other harmful proteins in GVBs may signify that aberrant signaling cascades are activated and that GVB-bearing cells are degenerating. Also prolonged activation of initially protective stress pathways like the UPR may contribute to neurodegeneration [117] (Table 3). Indeed, in the AD hippocampus, the main GVB hub, the levels of the UPR-induced transcription factor CHOP – a key mediator of the pro-apoptotic UPR signaling pathways – are increased [75]. It is unknown whether this increase in CHOP levels is specifically derived from the population of GVB-positive neurons. In a recent publication, levels of the ER proteins BiP, SigR1 and VAPB were found to be elevated in the AD compared to control hippocampus [148]. However, within the AD hippocampus, the levels of these proteins – quantified by means of the average pixel intensity within a neuron – were lower in neurons with abundant GVBs compared to neurons without GVBs. This quantitative approach to identify alterations in the population of GVB-containing neurons is commendable, although the area occupied by both GVBs and the concomitant tau pathology is a confounding factor in this method and needs further study. Nevertheless, these data may indicate differences in ER homeostasis between GVB-positive and GVB-negative neurons. In line with a detrimental fate of neurons with GVBs, possible “ghost GVBs” – named in reference to extracellular “ghost NFTs” that remain after the death of an NFT-bearing neuron – have been described in the aged human brain [47, 48]. Ghost GVBs were defined as deposits morphologically resembling GVBs but without a discernable living cell body or nucleus and were detected with an anti-tau or anti-pTDP43 antibody. This could indicate that at least a population of neurons with GVBs degenerates. Future studies using additional GVB and cellular markers as well as higher resolution microscopy should confirm the existence of ghost GVBs.

The GVB load negatively correlates with neuronal density in the AD hippocampus [7, 68]. Accordingly, the extent of GVBs is correlated with various measures of cognitive decline [32, 47, 48, 61, 68, 132]. However, these correlations are the result of the causal relation between tau pathology and GVB formation as in the same [32, 48, 68] and other [4, 34, 51, 98] studies tau pathology correlates similarly with neurodegeneration and symptom severity. Indeed, it was shown that GVBs are only associated with dementia when measures of tau pathology – the NFT Braak stage and cortical neuritic plaque pathology – are not controlled for in the analysis [48, 61]. The positive correlations between GVB load and neuropathological and clinical measures of disease severity can therefore be explained both by an active contribution of GVBs to cell death and by activation of the GVB response as a cellular defense mechanism in surviving neurons (Fig. 4). Clearly, further experiments are needed to unravel the functional implications of GVB formation, where the study of experimental models will be instrumental.

## Recommendations and concluding remarks

Although their first description dates back more than a century, GVBs are relatively underinvestigated. The recent discovery of tau pathology as the trigger for GVB formation has given an impulse to the GVB research field. This sets the stage for future research into the mechanism and downstream consequences of GVB formation in experimental models, but the finding of causality between tau pathology and GVB formation also has important implications for the interpretation of neuropathological data. Without the exclusion of concurrent tau pathology in patients with non-tau neurodegenerative disorders, no conclusions on a relation between other protein aggregates and GVBs can be drawn. Therefore, future neuropathological studies should always analyze the presence of comorbid tau pathology in GVB-bearing cells. Here, important progress can be made by studying the co-localization of GVBs and tau aggregates at the single cell level by double immunolabeling rather than using pathological staging. The likely possibility that minute amounts of pathological tau aggregates are sufficient to trigger GVB formation implies that the exclusion of tau comorbidity in GVB-containing neurons may be hampered by detection limitations. To this end, confocal microscopy is recommended that enables single cell analysis at high resolution. Furthermore, to reduce the probability of tau co-pathology, specifically those brain areas with disease-specific pathology should be studied. Importantly, definite proof of a causal relation between non-tau protein aggregation and GVB formation can only be obtained in experimental models. Based on the current human neuropathological data, α-synuclein and DPR protein aggregates are the prime candidates for putative non-tau GVB induction and they await experimental validation. To streamline future research, we have formulated recommendations for the validation of a GVB identity in tissue and cell models. These guidelines include immunopositivity for CK1δ and at least one additional common GVB marker as well as proof of the characteristic GVB morphology (Table 1; Fig. 1). In Tables 2 and 4, previous studies on GVBs in non-tau neurodegenerative proteinopathies, experimental models and aged animals are aligned with these refined criteria. In this rapidly changing field, potential new GVB markers are identified continuously and their GVB localization can also be rigorously validated following the proposed guidelines. Our recommendations for the identification of GVBs will help to systematically identify GVBs and thereby facilitate future research towards the elucidation of the role of these enigmatic structures in the pathogenesis of tauopathies and other neurodegenerative proteinopathies.

## Supplementary information

**Additional file 1: Supplementary Table 1.** Overview of primary antibodies commonly used to detect GVB.

## Abbreviations

Ab: Antibody
Aβ: Amyloid β
AD: Alzheimer’s disease
ALS: Amyotrophic lateral sclerosis
APC: Adenomatous polyposis coli
APP: Amyloid precursor protein
APP^Sw^: Mutant amyloid precursor protein K670M/N671L
APP^Sw,Ind^: Mutant amyloid precursor protein K670M/N671L/V717F
APP^Sw/L^: Mutant amyloid precursor protein K670M/N671L/V717I
α-syn: α-synuclein
AT8: Antibody recognizing tau phosphorylated at Ser202/Thr205
AT100: Antibody recognizing tau phosphorylated at Thr212/Ser214
AT270: Antibody recognizing tau phosphorylated at Thr181
BiP: Binding immunoglobulin protein
β-COP: Coatomer subunit β
CA: Cornu ammonis
c-Abl: Abelson tyrosine kinase
CBD: Corticobasal degeneration
CDK5: Cyclin-dependent kinase 5
CHMP2B: Charged multivesicular body protein 2B
CHOP: C/EBP homologous protein
CK1α: Casein kinase 1 α
CK1δ: Casein kinase 1 δ
CK1ε: Casein kinase 1 ε
CTSD: Cathepsin D
C9ORF72: Chromosome 9 open reading frame 72
DAB: 3.3′-diaminobenzidine
DAPI: 4′,6-diamidino-2-phenylindole
DPR: Dipeptide repeat
DQ-BSA: Self-quenched dye conjugate of bovine serum albumin
Dvl3: Segment polarity protein dishevelled homolog 3
EEA1: Early endosome antigen 1
eIF2α: Eukaryotic translation initiation factor 2α
EM: Electron microscopy
ER: Endoplasmic reticulum
FTLD: Frontotemporal lobar degeneration
FTLD/ALS-C9: TDP-43 related frontotemporal lobar degeneration / amyotrophic lateral sclerosis caused by a hexanucleotide repeat expansion in C9ORF72
FUS: Fused in sarcoma
GFP: Green fluorescent protein
GSK-3β: Glycogen synthase kinase-3β
GSS: Gerstmann-Sträussler-Scheinker syndrome
GVBs: Granulovacuolar degeneration bodies
GVD: Granulovacuolar degeneration
G3BP: Ras-GTPase-activating protein-binding protein
HEK293: Human embryonic kidney 293 cells
Hrd1: E3 ligase HMG-CoA reductase degradation protein 1
HNE: 4-hydroxy-2-nonenal
Htt: Huntingtin
H&E: Hematoxylin and eosin
iPSC: Induced pluripotent stem cell
IRE1α: Inositol-requiring enzyme 1α
LAMP1: Lysosome-associated membrane protein 1
LC3: Microtubule-associated protein 1A/1B-light chain 3
LIMP2: Lysosomal integral membrane protein 2
LRRK2: Leucine-rich repeat kinase 2
LSD: Lysosomal storage disorder
MAPT: Microtubule-associated protein tau
MARK3: Microtubule affinity regulating kinase isoform 3
MARK4: Microtubule affinity regulating kinase isoform 4
MLKL: Mixed lineage kinase domain-like protein
MPM-2: Ab antibody recognizing mitotic phospho-epitopes
MSA: Multiple system atrophy
M6PR: Mannose 6-phosphate receptor
NF: Neurofilament
NF-κB: Nuclear factor-kappa B
NFT: Neurofibrillary tangle
OspA: Outer surface protein of Borrelia burgdorferi spirochete
p: Phosphorylated
PERK: Protein kinase R (PKR)-like endoplasmic reticulum kinase
PGRN: Progranulin gene
PHF-1: Antibody recognizing tau phosphorylated at Ser396/Ser404
PICALM: Phosphatidylinositol-binding clathrin assembly protein
PKR: Double-stranded RNA-dependent protein kinase
PRKN: Parkin RBR E3 ubiquitin protein ligase
PPND: Pallido-ponto-nigral degeneration
PRNP: Prion protein gene
PrP: Prion protein
PrP^Sc^: PrP-Scrapie
PSKs: Prostate-derived sterile 20-like kinases
PSP: Progressive supranuclear palsy
PS1: Presenilin-1
PS1^dE9^: Mutant PS1 with deletion of exon 9
p300: E1A binding protein p300
p38: MAP kinase p38 mitogen-activated protein kinase
p62: Sequestosome 1
Rab7: Ras-related protein 7
Rab10: Ras-related protein Rab10
Rab11: Ras-related protein 11
RIPK1: Receptor-interacting serine/threonine-protein kinase 1
RIPK3: Receptor-interacting serine/threonine-protein kinase 3
SAPK/JNK: Stress-activated protein kinase/c-Jun N-terminal kinase
SigR1: Sigma receptor 1
SIL1: Nucleotide exchange factor SIL1
SMI-31: Antibody recognizing phosphorylated neurofilament H and M
SNCA: α-synuclein gene
SSBP1: Single stranded DNA binding protein 1
Syk: Spleen tyrosine kinase
S6 40S: Ribosomal subunit protein S6
TDP-43: TAR DNA-binding protein 43
TFEB: Transcription factor EB
Tg: Transgenic
TG3: Antibody recognizing tau phosphorylated at Thr231 (also conformation-specific)
TMEM230: Transmembrane protein 230
Ubi: Ubiquitin
UPR: Unfolded protein response
VAPB: Vesicle-associated membrane protein-associated protein B
VPS4a: Vacuolar protein sorting-associated protein 4A
