# Supplementary material for: Untangling the origin and function of granulovacuolar degeneration bodies in neurodegenerative proteinopathies
Source: Acta Neuropathol Commun. 2020 Sep 3;8:153. doi: 10.1186/s40478-020-00996-5 (PMC7469111; doi:10.1186/s40478-020-00996-5)
Supplement: Supplementary file 1 — Additional file 1: Supplementary Table 1. Overview of primary antibodies commonly used to detect GVB. [file 40478_2020_996_MOESM1_ESM.pdf]

Supplementary Table 1 Overview of primary antibodies commonly used to detect GVB

Target protein	Source	Product number/ clone	GVB labeling shown in		
			Human brain	Mouse brain	Primary mouse neurons
CK1δ	Icos	IC128A	[2, 3, 10, 15]		
CK1δ	Abcam	ab85320/AF12G4	[12, 19, 24, 25, 31, 34]	[11, 19, 34]	
CK1δ	Abcam	ab151793	[13, 34]	[34]	
CK1δ *	Abcam	ab37971	[17]		
CK1δ	Santa Cruz Biotechnology	sc-55553	[6, 8, 29]		[30]
CK1δ	ThermoScientific	PA5-32129	[30]	[30]	[30]
CK1δ	[26]	antiserum 108	[14, 27]		
CK1ε	BD Transduction Laboratories	40520	[3, 15]		
CK1ε *	Santa Cruz Biotechnology	sc-25423/H-60	[27]	[11]	[30]
CK1ε	[1]	antiserum 712	[14, 27]		
CHMP2B	Abcam	ab33174	[2, 19, 21, 32, 33], possibly [25]	[19]	[30]
pPERK (Thr981) *	Santa Cruz Biotechnology	sc-32577	[4, 18, 20, 23, 29, 30]	[11, 30]	[30]
pPERK (Thr980)	Cell Signaling	3191	[5], possibly: [28]		
pIRE1α (Ser724)	Novus Biologicals	NB100-2323	[4, 18, 23, 29]	[11, 30]	[30]
peIF2α (Ser52)	Sigma-Aldrich	E2152	[4]	[30]	
peIF2α (Ser51)	Cell Signaling	9721	Possibly: [18, 28]		[30]
peIF2α (Ser52) *	Santa Cruz Biotechnology	sc-101670		Possibly: [11]	
pTDP-43 (Ser409/410)	Cosmo Bio Co	TIP-PTD-M01/11-9	[8, 34]	[34]	
pTDP-43 (Ser409/410-2) *	Cosmo Bio Co	TIP-PTD-P02	[7, 12, 25, 27]		
pTDP-43 (Ser409/410)	[9]	2A	[9, 20]		
pTDP-43 (Ser409/410)	[22]	1D3	[16]		
pTDP-43 (Ser403/404) *	Cosmo Bio Co	TIP-PTD-P05	Possibly: [9]		
LAMP1	Abgent/Abcepta	AP1823a	[2]		
LAMP1	Abcam	ab25245			[30]
LIMP2	Novus Biologicals	NB400-129			[30]

Target protein, source and product/clone number of the primary antibody are listed. For phospho-specific antibodies, the phospho-epitope is indicated. Examples of references in which the antibody was used to detect GVBs in human or mouse tissue or primary mouse neurons with seeded tau pathology are listed. For some references, product number was deduced from description of species/clonality/isotype as indicated by *Possibly*; * antibody discontinued

References accompanying Supplementary Table 1

1. Brockschmidt C, Hirner H, Huber N, Eismann T, Hillenbrand A, Giamas G, et al. (2008) Anti-apoptotic and growth-stimulatory functions of CK1 delta and epsilon in ductal adenocarcinoma of the pancreas are inhibited by IC261 in vitro and in vivo. Gut 57:799–806. doi: 10.1136/gut.2007.123695

2. Funk KE, Mrak RE, Kuret J (2011) Granulovacuolar degeneration (GVD) bodies of Alzheimer's disease (AD) resemble late-stage autophagic organelles. Neuropathol Appl Neurobiol 37:295–306. doi: 10.1111/j.1365-2990.2010.01135.x

3. Ghoshal N, Smiley JF, DeMaggio AJ, Hoekstra MF, Cochran EJ, Binder LI, et al. (1999) A new molecular link between the fibrillar and granulovacuolar lesions of Alzheimer's disease. Am J Pathol 155:1163–1172. doi: 10.1016/S0002-9440(10)65219-4

4. Hoozemans JJM, van Haastert ES, Nijholt DAT, Rozemuller AJM, Eikelenboom P, Scheper W (2009) The unfolded protein response is activated in pretangle neurons in Alzheimer's disease hippocampus. Am J Pathol 174:1241–1251. doi: 10.2353/ajpath.2009.080814

5. Hoozemans JJM, Veerhuis R, Van Haastert ES, Rozemuller JM, Baas F, Eikelenboom P, et al. (2005) The unfolded protein response is activated in Alzheimer's disease. Acta Neuropathol 110:165–172. doi: 10.1007/s00401-005-1038-0

6. Hou X, Fiesel FC, Truban D, Castanedes Casey M, Lin W-I, Soto AI, et al. (2018) Age- and disease-dependent increase of the mitophagy marker phospho-ubiquitin in normal aging and Lewy body disease. Autophagy 14:1404–1418. doi: 10.1080/15548627.2018.1461294

7. Hunter S, Hokkanen SRK, Keage HAD, Fleming J, Minett T, Polvikoski T, et al. (2020) TDP-43 related neuropathologies and phosphorylation state: associations with age and clinical dementia in the Cambridge City over-75s Cohort. J Alzheimers Dis 75:337–350. doi: 10.3233/JAD-191093

8. Iyer AM, van Scheppingen J, Milenkovic I, Anink JJ, Adle-Biassette H, Kovacs GG, et al. (2014) mTOR hyperactivation in Down syndrome hippocampus appears early during development. J Neuropathol Exp Neurol 73:671–683. doi: 10.1097/NEN.0000000000000083

9. Kadokura A, Yamazaki T, Kakuda S, Makioka K, Lemere CA, Fujita Y, et al. (2009) Phosphorylation-dependent TDP-43 antibody detects intraneuronal dot-like structures showing morphological characters of granulovacuolar degeneration. Neurosci Lett 463:87–92. doi: 10.1016/j.neulet.2009.06.024

10. Kannanayakal TJ, Tao H, Vandre DD, Kuret J (2006) Casein kinase-1 isoforms differentially associate with neurofibrillary and granulovacuolar degeneration lesions. Acta Neuropathol 111:413–421. doi: 10.1007/s00401-006-0049-9

11. Köhler C, Dinekov M, Götz J (2014) Granulovacuolar degeneration and unfolded protein

response in mouse models of tauopathy and Aβ amyloidosis. Neurobiol Dis 71:169–179. doi: 10.1016/j.nbd.2014.07.006

12. Koper MJ, Van Schoor E, Ospitalieri S, Vandenberghe R, Vandenbulcke M, von Arnim CAF, et al. (2020) Necrosome complex detected in granulovacuolar degeneration is associated with neuronal loss in Alzheimer's disease. Acta Neuropathol 139:463–484. doi: 10.1007/s00401-019-02103-y

13. Kork F, Jankowski J, Goswami A, Weis J, Brook G, Yamoah A, et al. (2018) Golgin A4 in CSF and granulovacuolar degenerations of patients with Alzheimer disease. Neurology 91:e1799–e1808. doi: 10.1212/WNL.0000000000006457

14. Kumar S, Wirths O, Stüber K, Wunderlich P, Koch P, Theil S, et al. (2016) Phosphorylation of the amyloid β-peptide at Ser26 stabilizes oligomeric assembly and increases neurotoxicity. Acta Neuropathol 131:525–537. doi: 10.1007/s00401-016-1546-0

15. Lagalwar S, Berry RW, Binder LI (2007) Relation of hippocampal phospho-SAPK/JNK granules in Alzheimer's disease and tauopathies to granulovacuolar degeneration bodies. Acta Neuropathol 113:63–73. doi: 10.1007/s00401-006-0159-4

16. Lippa CF, Rosso AL, Stutzbach LD, Neumann M, Lee VM-Y, Trojanowski JQ (2009) Transactive response DNA-binding protein 43 burden in familial Alzheimer disease and Down syndrome. Arch Neurol 66:1483–1488. doi: 10.1001/archneurol.2009.277

17. Lund H, Gustafsson E, Svensson A, Nilsson M, Berg M, Sunnemark D, et al. (2014) MARK4 and MARK3 associate with early tau phosphorylation in Alzheimer's disease granulovacuolar degeneration bodies. Acta Neuropathol Commun 2:22. doi: 10.1186/2051-5960-2-22

18. Makioka K, Yamazaki T, Fujita Y, Takatama M, Nakazato Y, Okamoto K (2010) Involvement of endoplasmic reticulum stress defined by activated unfolded protein response in multiple system atrophy. J Neurol Sci 297:60–65. doi: 10.1016/j.jns.2010.06.019

19. Midani-Kurçak JS, Dinekov M, Puladi B, Arzberger T, Köhler C (2019) Effect of tau-pathology on charged multivesicular body protein 2b (CHMP2B). Brain Res 1706:224–236. doi: 10.1016/j.brainres.2018.11.008

20. Nagamine S, Yamazaki T, Makioka K, Fujita Y, Ikeda M, Takatama M, et al. (2016) Hypersialylation is a common feature of neurofibrillary tangles and granulovacuolar degenerations in Alzheimer's disease and tauopathy brains. Neuropathology 36:333–345. doi: 10.1111/neup.12277

21. Nakamori M, Takahashi T, Yamazaki Y, Kurashige T, Yamawaki T, Matsumoto M (2012) Cyclin-dependent kinase 5 immunoreactivity for granulovacuolar degeneration. NeuroR 23:867–872. doi: 10.1097/WNR.0b013e328358720b

22. Neumann M, Kwong LK, Lee EB, Kremmer E, Flatley A, Xu Y, et al. (2009) Phosphorylation of S409/410 of TDP-43 is a consistent feature in all sporadic and familial forms of TDP-43 proteinopathies. Acta Neuropathol 117:137–149. doi: 10.1007/s00401-008-0477-9

23. Nijholt DAT, van Haastert ES, Rozemuller AJM, Scheper W, Hoozemans JJM (2012) The unfolded protein response is associated with early tau pathology in the hippocampus of tauopathies. J Pathol 226:693–702. doi: 10.1002/path.3969

24. Nishikawa T, Takahashi T, Nakamori M, Hosomi N, Maruyama H, Miyazaki Y, et al. (2016) The identification of raft-derived tau-associated vesicles that are incorporated into immature tangles and paired helical filaments. Neuropathol Appl Neurobiol 42:639–653. doi: 10.1111/nan.12288

25. Riku Y, Duyckaerts C, Boluda S, Plu I, Le Ber I, Millecamps S, et al. (2019) Increased prevalence of granulovacuolar degeneration in C9orf72 mutation. Acta Neuropathol 138:783–793. doi: 10.1007/s00401-019-02028-6

26. Stöter M, Bamberger A-M, Aslan B, Kurth M, Speidel D, Löning T, et al. (2005) Inhibition of casein kinase I delta alters mitotic spindle formation and induces apoptosis in trophoblast cells. Oncogene 24:7964–7975. doi: 10.1038/sj.onc.1208941

27. Thal DR, Del Tredici K, Ludolph AC, Hoozemans JJM, Rozemuller AJ, Braak H, et al. (2011) Stages of granulovacuolar degeneration: their relation to Alzheimer's disease and chronic stress response. Acta Neuropathol 122:577–589. doi: 10.1007/s00401-011-0871-6

28. Unterberger U, Höftberger R, Gelpi E, Flicker H, Budka H, Voigtländer T (2006) Endoplasmic reticulum stress features are prominent in Alzheimer disease but not in prion diseases in vivo. J Neuropathol Exp Neurol 65:348–357. doi: 10.1097/01.jnen.0000218445.30535.6f

29. Wiersma VI, van Hecke W, Scheper W, van Osch MAJ, Hermsen WJM, Rozemuller AJM, et al. (2016) Activation of the unfolded protein response and granulovacuolar degeneration are not common features of human prion pathology. Acta Neuropathol Commun 4:113. doi: 10.1186/s40478-016-0383-7

30. Wiersma VI, van Ziel AM, Vazquez-Sanchez S, Nölle A, Berenjeno-Correa E, Bonaterra-Pastra A, et al. (2019) Granulovacuolar degeneration bodies are neuron-selective lysosomal structures induced by intracellular tau pathology. Acta Neuropathol 138:943–970. doi: 10.1007/s00401-019-02046-4

31. Yamaguchi Y, Ayaki T, Li F, Tsujimura A, Kamada M, Ito H, et al. (2019) Phosphorylated NF-κB subunit p65 aggregates in granulovacuolar degeneration and neurites in neurodegenerative diseases with tauopathy. Neurosci Lett 704:229–235. doi: 10.1016/j.neulet.2019.03.036

32. Yamazaki Y, Matsubara T, Takahashi T, Kurashige T, Dohi E, Hiji M, et al. (2011) Granulovacuolar degenerations appear in relation to hippocampal phosphorylated tau accumulation in various neurodegenerative disorders. PLoS One 6:e26996. doi: 10.1371/journal.pone.0026996

33. Yamazaki Y, Takahashi T, Hiji M, Kurashige T, Izumi Y, Yamawaki T, et al. (2010) Immunopositivity for ESCRT-III subunit CHMP2B in granulovacuolar degeneration of neurons in the Alzheimer's disease hippocampus. Neurosci Lett 477:86–90. doi: 10.1016/j.neulet.2010.04.038

34. Yamoah A, Tripathi P, Sechi A, Köhler C, Guo H, Chandrasekar A, et al. (2020) Aggregates of RNA binding proteins and ER chaperones linked to exosomes in granulovacuolar degeneration of the Alzheimer's disease brain. J Alzheimer's Dis 75:139–156. doi: 10.3233/jad-190722
